# A Stepwise Preclinical Microvascular Training Model Based on the Superficial Inferior Epigastric Vessel Flap in the Rat: A Pilot Study

**DOI:** 10.3390/medicina62101963

**Published:** 2026-10-11

**Authors:** Florin-Vlad Hodea, Stefan Cacior, Andreea-Maria Fira, Vladut-Alin Ratoiu, Eliza-Maria Bordeanu-Diaconescu, Ioan Lascar, Andreea Grosu-Bularda

**Affiliations:** 1 Department 11—Discipline of Plastic and Reconstructive Surgery, “Carol Davila” University of Medicine and Pharmacy, 050474 Bucharest, Romania; florin-vlad.hodea@drd.umfcd.ro (F.-V.H.); andreea-maria.fira0720@stud.umfcd.ro (A.-M.F.); vladut-alin.ratoiu@rez.umfcd.ro (V.-A.R.); ioan.lascar@umfcd.ro (I.L.); andreea.grosu-bularda@umfcd.ro (A.G.-B.); 2 Clinic of Plastic Surgery, Aesthetic and Reconstructive Microsurgery, Bucharest Clinical Emergency Hospital, 014461 Bucharest, Romania; eliza.diaconescu@umfcd.ro

**Keywords:** microsurgery, training, free flap, superficial inferior epigastric flap, rat model, microvascular anastomosis, supermicrosurgery, surgical education

## Abstract

*Background and Objectives*: Transitioning from non-living simulation to clinical free-tissue transfer remains challenging. This pilot study aimed to develop and evaluate a staged in vivo microsurgical training protocol based on the rat superficial inferior epigastric (SIE) flap, in which the anatomical substrate is held constant and only technical demand varies between stages. *Materials and Methods*: Twenty male Wistar rats were used in five stages of increasing difficulty, progressing from orthotopic replantation and contralateral inguinal transfer to cervical transfer with end-to-end and end-to-side anastomoses using different donor vessel preparations. Outcomes were immediate patency, patency at 10 min, anastomotic leakage, procedural success (immediate patency without moderate-to-severe leakage) and anastomosis time. Proportions are reported with Wilson 95% confidence intervals; groups were compared with Fisher’s exact test, trends across stages with the Cochran–Armitage test, and individual anastomosis times with separate arterial and venous linear regressions on stage number, using HC3 confidence intervals unadjusted for within-animal clustering. *Results*: Fifty-six anastomoses were performed (30 arterial, 26 venous). Immediate patency was 83.3% (25/30) for arterial and 61.5% (16/26) for venous anastomoses, patency at 10 min 70.0% and 46.2%, and procedural success 80.0% and 57.7% (*p* = 0.087). The arterial–venous difference was of similar magnitude at every endpoint (21.8, 23.8 and 22.3 percentage points) and was already established at declamping: among initially patent anastomoses, attrition by 10 min was 16.0% for arteries and 25.0% for veins (*p* = 0.69). No significant trend across stages was detected for success (arterial *p* = 0.38; venous *p* = 0.94) or patency. Individual anastomosis time increased with stage, with slopes of 2.08 min per stage for arteries (95% HC3 CI 1.46–2.71; R^2^ = 0.703) and 2.69 min per stage for veins (95% HC3 CI 2.18–3.19; R^2^ = 0.821). Mean venous time was higher at every stage. *Conclusions*: Even considering the limitations of the present study, the staged SIE flap model provides a graded, quantifiable, and standardised framework for preclinical free flap training within a single anatomical substrate.

## 1. Introduction

Microsurgery can be defined as the set of surgical gestures performed under the magnification of an operating microscope, using instruments and suture materials adapted to the small dimensions of the structures approached [1]. Although the concept emerged at the beginning of the twentieth century, it became popular among surgeons only after 1960, when anastomoses of the blood vessels of the limbs, including the digits, became feasible [1]. In 1973, Daniel and Taylor performed the first free tissue transfer using a groin flap based on the superficial inferior epigastric artery, and free flaps thus came closer to becoming a new field of interest for microsurgery [2].

The usage of free flaps allowed complex trauma, post-oncological resection defects and deforming congenital anomalies to be approached from a new perspective, restoring both the appearance and the function of the affected structures. This versatility has significantly improved the results of reconstructive surgery and allowed a superior quality of life for patients [2,3,4]. The continuous adaptation of microsurgery to increasing demands for faithful restoration of the human body has generated a need for additional training that cannot be obtained solely by attending microsurgical interventions in the operating room [5,6]. Acquiring these techniques requires dexterity and continuous practice, both obtained through training on models that simulate the different steps of microsurgical procedures [7].

The structures approached in microsurgery are delicate, so that even minor errors may have major consequences. In addition, practicing techniques during live surgery creates a stressful environment for young surgeons, many of whom lack the confidence necessary to perform certain steps under the operating microscope [8,9]. All of these factors lead to a slow and demanding learning curve, which can be overcome only through constant and extensive preparation [10]. Failure of a microsurgical anastomosis may compromise a free flap or a replantation; this is one of the reasons why practical training has moved outside the operating theatre, into laboratories specifically equipped to provide the conditions required for microsurgical practice [11]. In such an environment, training may take place in a non-stressful setting that allows gradual, staged preparation, the surgeon being able to simulate almost every step of an operation, depending on the model used [12].

Neuropsychological learning patterns show that the acquisition of new information, particularly motor skills, occurs in two phases: a first phase characterised by rapid assimilation, and a second, plateau phase in which the acquisition of new techniques is minimal but automation and ease of movement continue to improve [13]. The usefulness of laboratory microsurgical training has been analysed and demonstrated in numerous studies over the last two decades. Repeated practice of carotid artery revascularization in nine rats showed a 54 min difference between the initial and the final operative time (*p* = 0.001) [14]. In another study, residents classified as microsurgical novices performed anastomoses on silicone tubes; residents who had not attended a laboratory training course did not modify any of the 14 measured parameters, whereas those who practiced improved 10 parameters (*p* < 0.05) compared with their own baseline and reached values statistically similar to those of experts for two parameters (*p* < 0.05) [15].

Training models can be classified, according to the difficulty of their use and their ability to reproduce the operating-room environment, into non-biological models (latex gloves, silicone tubes), non-living tissue models (chicken wings, placenta) and living tissue models (rodents, pigs) [16,17]. Among these, living animals offer the closest experience to that of the operating theatre, allowing patency to be assessed not only immediately but also after a period of time, so that phenomena such as thrombosis can be observed [18]. Staging microsurgical training, starting from simple synthetic models and progressing to experimental models, has proved useful, and several centers have developed their own training programmes for young surgeons [19,20,21,22]. Besides the advantage of acquiring microsurgical techniques in a logical order, beginning with easily accessible and inexpensive materials, this approach reduces the number of live animals used, which are reserved for the final part of training, where advanced techniques are practiced [19].

The rat is frequently described as the ideal specimen for living microsurgical training. Its small size, the multitude of available vessels, the ease of access and the presence of hemodynamics explain why it is so often used in this field [23]. The most reproducible model simulating microsurgical procedures on patients is considered to be the live rat, which is suitable not only for basic training but also for practicing free flap transfer, replantation and lymphatic or lympho-venous anastomoses [24]. Its blood vessels have millimetric or even submillimetric calibers, smaller than human vessels, which allows technically more demanding operative steps to be performed [25]. Beyond the femoral vessels, the rat offers many other vessels of interest, such as the common carotid artery, with a diameter below 1 mm, or the infrarenal abdominal aorta, with a larger diameter, allowing several anatomical regions to be approached and a variety of practical exercises to be undertaken [26]. The types of anastomoses that can be performed on this model are numerous, ranging from end-to-end and end-to-side to side-to-side configurations [27,28].

In 1963, Goldwyn described the first microsurgically transferred flap, in a canine model, based on the superficial epigastric vessels [29]. In 1967, Strauch and Murray described a similar flap in a rat model, which proved far easier to use [30,31]. The epigastric flap can be transferred ipsilaterally, contralaterally or to the cervical region. In Wistar rats, this flap has several advantages, including a low morbidity rate, the possibility of primary closure of the donor site, and a recipient region, the cervical area, with the lowest chance of self-mutilation [32,33]. The femoral vessels pass beneath the inguinal ligament and give rise to the superficial inferior epigastric vessels, which supply the lower part of the abdominal wall; these vessels reach a mean caliber of 0.5 mm for the artery and 0.7 mm for the vein [34]. Transfer of the SIE flap to the cervical region is frequently used for practicing the free tissue transfers performed in clinical practice: the surgeon can learn and refine the operative steps and plan a future reconstructive procedure [35]. Performing microsurgical dissection in two regions (the inguinal fossa and the neck) and completing two technically and anatomically demanding anastomoses, make this flap a valuable experience in microsurgical practice [36].

One of the key strengths of the rat model, which coincides with the scope of this study, is the possibility of building a staged algorithm progressing from simple to difficult performance steps of free tissue transfers in a non-human living model. In the present study we propose a staged microsurgical training programme, designed to develop the competences required for free tissue transfer in a realistic in vivo environment but with minimal risk to patients.

## 2. Materials and Methods

This experimental pilot study was carried out in compliance with the current ethical regulations and was approved by the Ethics Committee of the Bucharest Clinical Emergency Hospital, registration number 2582 of 26 March 2024. All experimental procedures were conducted after ethical approval had been granted on 26 March 2024.

A completed ARRIVE 2.0 reporting checklist, identifying the manuscript locations addressing each item and explaining any unavailable information, is provided as Supplementary Checklist S1.

The study was designed to elaborate an algorithm for the staged completion of microvascular training steps on a rat flap based on the superficial inferior epigastric vessels, with minimal sacrifice of live subjects.

The rationale for the model is the following. The transition from training on non-living models to transfer in the human patient is, in most cases, difficult, and predisposes to technical errors that may jeopardise the success of the anastomosis. The living rat model occupies this intermediate gap in training, making the transition to clinical microsurgery easier and safer. The difficulty offered by these experimental interventions is superior, since the maximum dimensions of rat vessels is comparable to that of human digital arteries at the level of the phalanges, which are among the smallest vessels handled microsurgically. In addition to facilitating this transition, the protocol does so in a staged, controlled manner, by levels of difficulty, from a simple femoral–femoral anastomosis up to the free transfer of the inguinal flap to the cervical region. This anatomical positioning of the flap allows easy monitoring of viability and prevents the animal from self-mutilating.

The laboratory animal chosen for this study was the Wistar rat, because of its frequent use in numerous experimental surgical studies, its availability, and the low cost of housing and maintenance. Twenty male Wistar rats weighing between 250 and 350 g were used. The animals were purchased by one of the authors from Laboratorul Biobază, Institutul Oncologic “Prof. Dr. Alexandru Trestioreanu”, Bucharest, Romania. The supplier identified the animals as Wistar rats. Formal documentation confirming their stock identity and a more specific stock designation were not available; therefore, the Wistar designation used in this study is based on supplier identification. They were kept under standard housing conditions, in spacious cages with a maximum of four animals per cage, with a diet appropriate for rodents and water ad libitum. The experiments were performed in the Microsurgery Laboratory of the Bucharest Clinical Emergency Hospital by a single operator, a sixth-year medical student with an estimated three years of experience in microsurgical training at hands-on workshops and in the laboratory. Practice consisted of non-living training models such as gloves, silicone tubes, flower petals, and chicken thigh vessels, with approximately 50 anastomoses performed in total prior to this study. The operator had minimal prior experience with live microsurgical training models, limited to basic microsurgical vessel anastomoses in rats, but no prior experience with free tissue transfers in live animals and no clinical operative experience. This background characterises the operator’s prior training; however, the fixed sequence of stages does not allow the effects of task difficulty and concurrent skill acquisition to be separated.

The experimental lot presented in this study does not overlap with any of the previous studies conducted by our team and was used solely for the purposes of this research.

Magnification was obtained using one tabletop Zeiss OPMI microscope. Suturing and fine dissection were performed under a ×25 magnification, while general dissection and knot tying were performed under a ×10 magnification.

In addition to the operating microscope, the following conventional surgical instruments were used: scalpel handle, surgical forceps, anatomical forceps, blunt-tipped dissecting scissors and sharp-tipped scissors. The microsurgical set comprised a curved-tip microvascular needle holder without a locking mechanism, fine-tipped forceps, curved round-tipped microsurgical scissors for dissection, straight sharp-tipped microsurgical scissors for adventitiectomy, microvascular approximators consisting of two metal clips mounted on a bar, and single microvascular metal clips.

Consumables included examination and sterile gloves, sterile drapes, sterile gauze, and 1 mL and 5 mL syringes. Solutions and anaesthetic agents comprised 0.9% sodium chloride, ketamine (100 mg/mL), xylazine (100 mg/mL), and sevoflurane. Because all procedures were terminal, skin closure was not performed. Monofilament nylon sutures (ETHILON, Ethicon) were used throughout. Ligations were performed with 4-0 sutures supplied with 19 mm reverse-cutting needles. Anastomoses involving the femoral and cervical vessels were performed with 8-0 and 9-0 sutures mounted on 6.5 mm taper-point needles. Anastomoses between the superficial inferior epigastric vessels and the cervical recipient vessels were performed with 10-0 sutures mounted on 3.8 mm taper-point needles.

Food was withheld for 12 h before surgery, while water remained available ad libitum. Each animal was weighed preoperatively to calculate the anaesthetic dose, and the sterile operative field was prepared with conventional and microsurgical instruments.

Anaesthesia was induced by inhalation of sevoflurane, followed by intraperitoneal administration of a ketamine–xylazine mixture used for anaesthesia and intraoperative analgesia. The mixture contained 8 mL of 0.9% sodium chloride, 1.75 mL of ketamine (100 mg/mL), and 0.25 mL of xylazine (100 mg/mL), yielding a total volume of 10 mL. The final concentrations were 17.5 mg/mL ketamine and 2.5 mg/mL xylazine. The mixture was administered at 0.1 mL per 20 g body weight (5 mL/kg), corresponding to initial doses of 87.5 mg/kg ketamine and 12.5 mg/kg xylazine.

The animals were monitored continuously by clinical observation of spontaneous movements, whisker movements, and respiratory patterns. The appearance of movements and an increase in respiratory rate were interpreted as signs of lightening anaesthesia and prompted intraperitoneal administration of an additional intraperitoneal injection of 0.5 mL of the same ketamine–xylazine mixture. Supplementary dosing was sometimes required during procedures lasting longer than 60 min.

Thermal support was provided throughout the procedure by covering the animals with sterile cloths and using containers filled with warmed saline as external heat sources. No additional fluid support was administered.

All procedures were terminal, with no planned recovery from anaesthesia. Following completion of the assessment of anastomotic patency and leakage at 10 min after declamping, euthanasia was performed by exsanguination under continued ketamine–xylazine anaesthesia and analgesia. Before exsanguination, ongoing cardiac activity and visible vascular pulsations were observed clinically. Death was confirmed clinically by the sustained absence of detectable cardiac activity, spontaneous respiration, and visible vascular pulsations throughout a 10 min observation period. No unplanned deaths occurred before scheduled euthanasia, and no animals were excluded from the study.

Hair was removed by applying a depilatory cream to the areas of interest, followed by rinsing under running water and, when necessary, the use of a disposable razor blade. The rat was placed on the operating table in the supine position, with the limbs abducted and restrained under minimal tension. The exposed skin was then antisepticised and the preoperative markings were drawn.

For the donor site, a rectangle centered on the inguinal region was drawn 2 cm from the midline. Its lower border lay immediately below the inguinal fold, the longitudinal side measuring 3 cm and the transverse side 2 cm. The rectangle corresponding to the recipient site was marked above the sternum, between the sternohyoid and sternocleidomastoid muscles, with a longitudinal axis of 2 cm from the level of the sternum and a transverse axis of 2.5 cm.

The skin incision was performed according to the preoperative drawing in the inguinal region of the subject. After complete division of the skin and subcutaneous tissue, the fat pad was exposed; the superficial inferior epigastric vessels lie in its depth, and the femoral vessels lie beneath it. Dissection of this fat pad was continued under the magnification of the operating microscope, using fine microsurgical instruments.

Using curved scissors and forceps, division of the fat pad was started in the medial, caudal region and continued towards the cranial, lateral region. After complete detachment, the pad was placed on a gauze kept moist constantly with saline solution. In the event of significant bleeding from the collateral vasculature of the fat pad, haemostasis was obtained by compression with a gauze or, if this was ineffective, by ligation of the responsible vessel.

**Figure 1:**
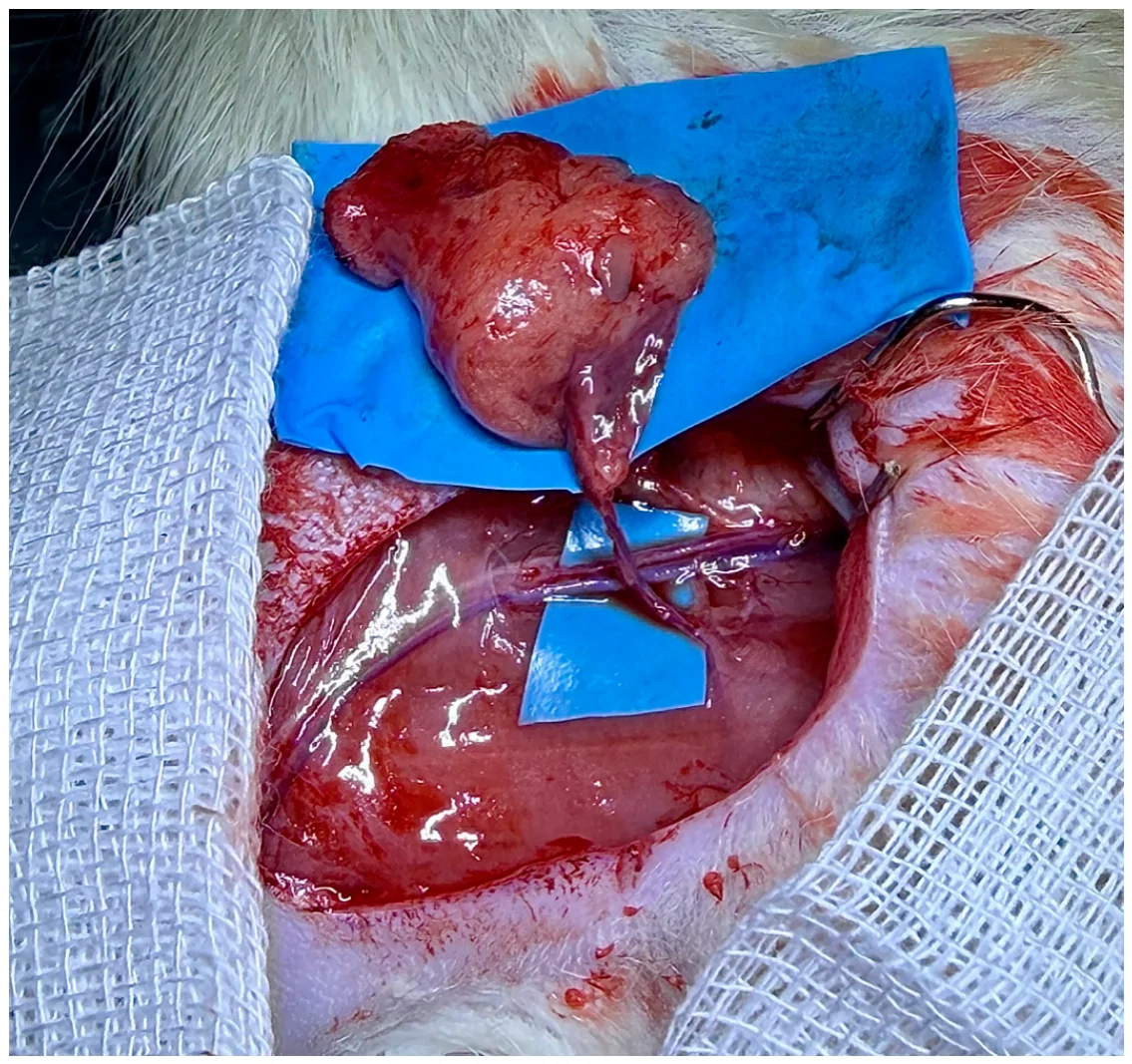
Visualisation of the femoral neurovascular bundle and of the epigastric vessels after placement of a retractor.

Beneath the fat pad, the femoral neurovascular bundle was exposed distal to the origin of the superficial inferior epigastric vessels. In order to visualize the proximal portion as well, a retractor was placed to draw the abdominal musculature medially (Figure 1). When correctly placed, this maneuver exposes the inguinal ligament, a firm, glossy, pearly-white structure from beneath which the femoral vessels emerge.

After flap dissection, the next step was to divide the septa derived from the deep fascia, in order to improve visibility of the working plane. The femoral neurovascular bundle is composed, from caudal to cranial, of the femoral vein, artery and nerve. The vessels are enveloped in a common sheath derived from the deep abdominal fascia. This sheath must be removed carefully in order to limit vessel injury, most frequently punctures leading to variable degrees of haemorrhage depending on the size of the defect and the type of vessel.

The individual sheaths of the femoral artery and vein were then removed, with abundant irrigation to eliminate connective or even adipose tissue remaining in the working field. The vein is the most caudal element of the bundle and has a thin, collapsible wall that flattens rapidly when handling and a larger diameter than the artery. The artery is pink-red, has a thicker, elastic wall and shows visible pulsations. The femoral nerve is not enveloped by the common sheath but adheres to the bundle through condensations of connective tissue at the border with the femoral artery; it is white or slightly yellowish, discreetly pearly and firm, and coarse handling may trigger reflexes such as extension of the hind limb.

**Figure 2:**
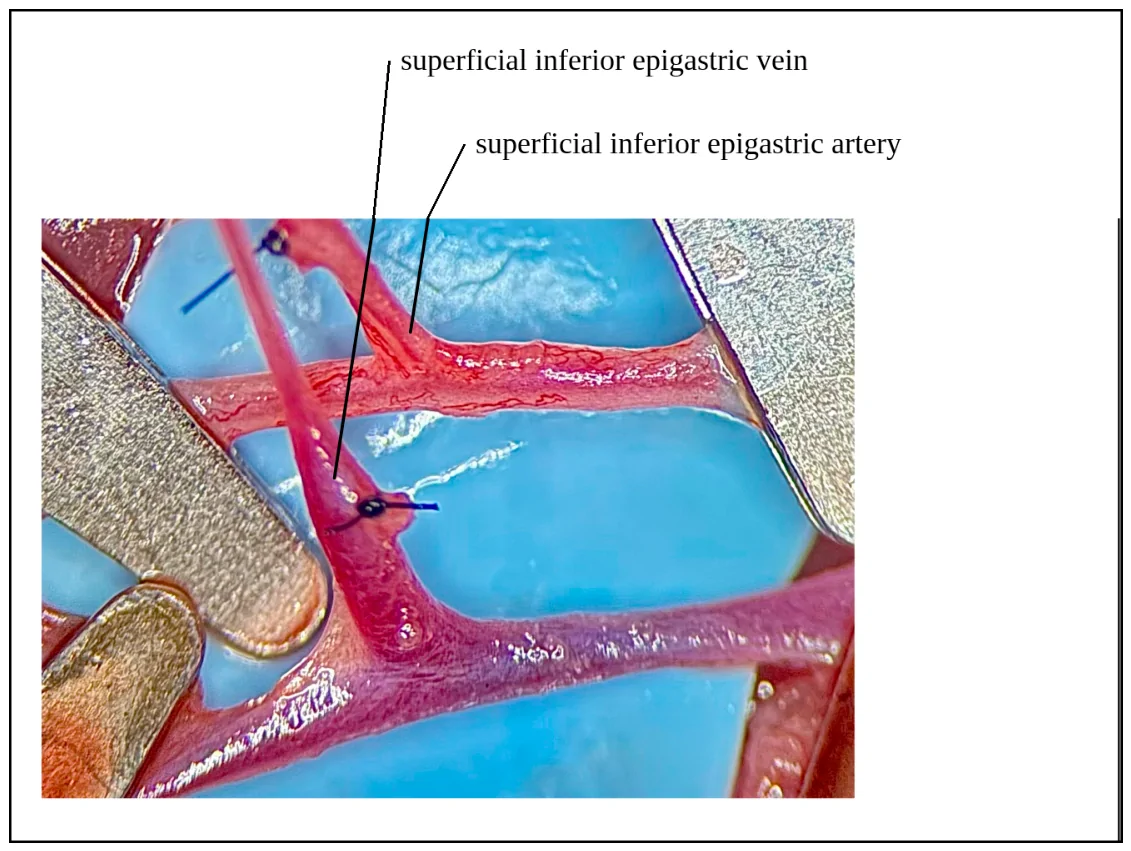
Origin of the superficial inferior epigastric vessels from the femoral vessels (microscopic image).

At the origin of the superficial inferior epigastric vessels (Figure 2), dissection must be performed carefully in order not to injure them. Alongside the vessels there are fine nerve filaments, or even a small independent nerve, which must be divided to allow flap mobilization.

Ligation of the lateral circumflex iliac artery and vein, thin branches that may connect with the superficial inferior epigastric vessels, was frequently required. Dissection was considered complete when the femoral vessels could be mobilised easily, without further connections to the deep planes, and when the epigastric vessels were separated and cleared of the connective tissue of the fat pad. The distal part of the pad was preserved.

The experimental protocol involved end-to-end and end-to-side arterial anastomoses and end-to-end venous anastomoses, using vessels of the inguinal region (femoral and superficial inferior epigastric arteries and veins) and of the cervical region (carotid artery and external jugular vein).

The femoral vessels were clamped, leaving sufficient space between the clips to allow harvest of the epigastric flap and to ensure adequate length of the femoral vascular stumps for a subsequent anastomosis. In the cervical region, the carotid artery was isolated and clamped proximally and distally, and the external jugular vein was ligated distally and clamped proximally.

After the arteriotomies, the vascular stumps were irrigated with saline to remove coagulated blood and cellular debris. Adventitiectomy was then performed, circumferentially for the arteries and only at the necessary points for the veins, followed by vascular dilatation, this last step ensuring congruence of the vascular stumps to be anastomosed. Removal of the adventitia is essential because this layer lacks the antithrombotic properties of the endothelium; exposure of collagen allows von Willebrand factor to bind and act as a bridge between collagen and platelets through the GPIb receptor, initiating platelet adhesion, while tissue factor activates the extrinsic coagulation pathway. The presence of adventitia in the lumen or at the suture line therefore significantly increases the risk of anastomotic thrombosis [37].

For end-to-end arterial or venous anastomoses, eight equidistant sutures were placed using the biangulation technique, the first stitches being made at 6 and 12 o’clock. The anterior wall was then sutured by the halving technique, followed by the posterior wall in the same manner [38]. For end-to-side arterial anastomoses, eight equidistant sutures were placed, with the placement of the first two sutures at equal distances on either side of the axis of the carotid artery. Because the vascular arrangement does not allow rotation of the vessels, the posterior wall was sutured first, by the halving technique, followed by the anterior wall.

Finally, the applied clips were removed sequentially, first the distal and then the proximal ones, in order to allow retrograde blood, with a lower flow than the anterograde flow, to come into contact with the anastomosis, to trigger physiological hemostasis and thus to seal the gaps in the vascular wall. Ten minutes after declamping, patency was checked by observing transverse blood pulsations distal to the anastomosis and by the Acland test, which consists of careful spreading of the vessel with a forceps, followed by compression of the retrograde flow and observation of the speed and degree of vascular filling by the blood passing through the anastomosis.

**Figure 3:**
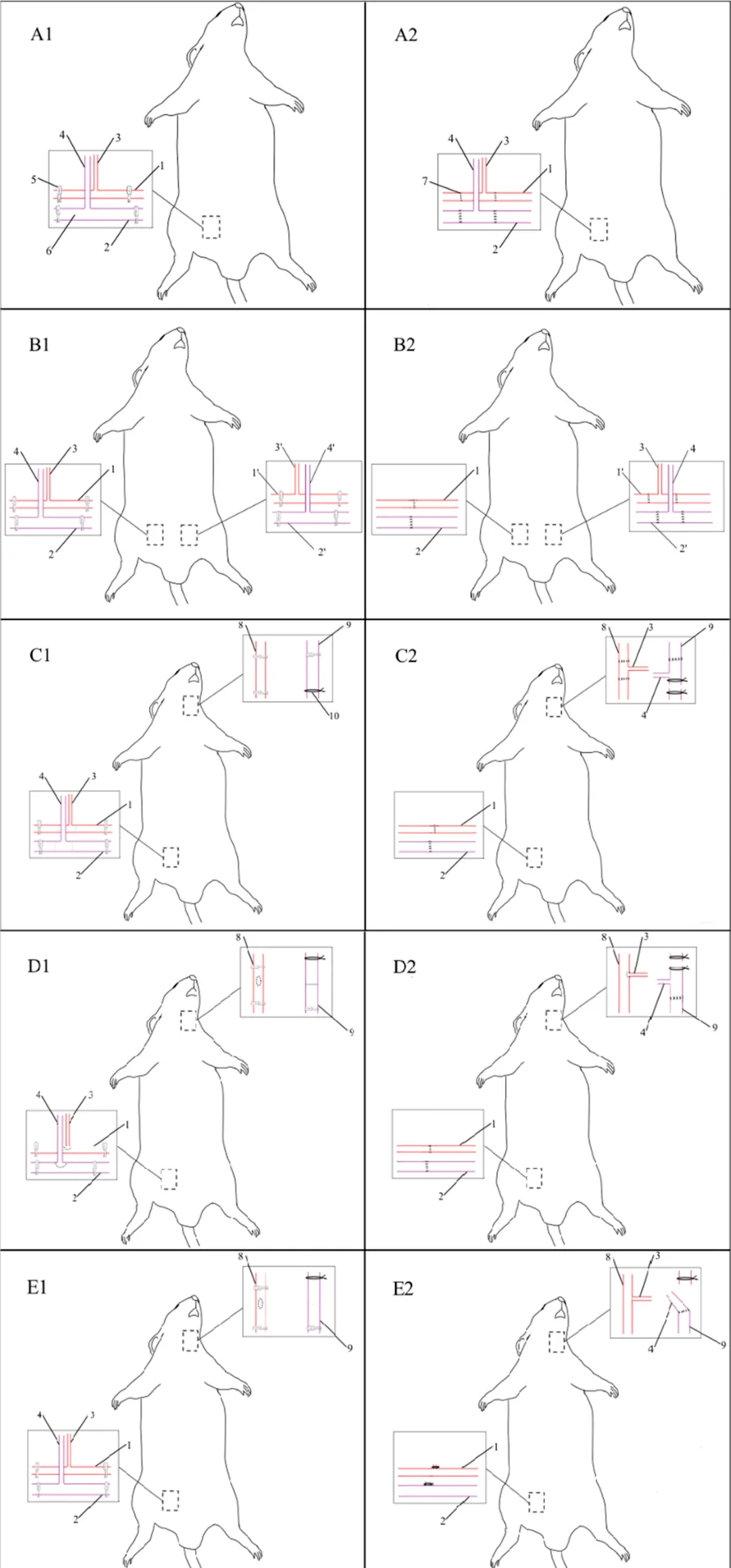
Illustrations of the operative steps according to stage (Stage I: (**A1**) preoperatively, (**A2**) postoperatively; Stage II: (**B1**) preoperatively, (**B2**)**:** post-operatively; Stage III: (**C1**) preoperatively, (**C2**) postoperatively; Stage IV: (**D1**) preoperatively, (**D2**) postoperatively; Stage V: (**E1**) preoperatively, (**E2**) postoperatively). Subscript 1 following the capital letter represents an intermediate step of the respective stage, and subscript 2 shows the final result. Key: 1 = right femoral artery; 1′ = left femoral artery; 2 = right femoral vein; 2′ = left femoral vein; 3 = right superficial inferior epigastric artery; 3′ = left superficial inferior epigastric artery; 4 = right superficial inferior epigastric vein; 4′ = left superficial inferior epigastric vein; 5 = vascular clip; 6 = arteriotomy; 7 = vascular anastomosis; 8 = left carotid artery; 9 = left external jugular vein; 10 = vascular ligature.

The experimental protocol was divided into five successive stages with progressive levels of difficulty (Figure 3). These involved mobilization of a flap based on the superficial epigastric vessels, transferred either to the inguinal region, ipsilaterally or contralaterally, or to the cervical region. The basic skills required for the experiment, such as handling of blood vessels and eye–hand coordination, had been acquired before these stages, on non-living models by the performing surgeon. The preparatory phase included femoral end-to-end arterial and venous anastomoses as well as end-to-side arterio-venous anastomoses in the inguinal region.

The experimental stages were performed in a fixed ascending order, from Stage I to Stage V, without randomisation of stage order. Subjects underwent procedures sequentially, with all procedures within a given stage completed before the subsequent stage commenced. Subjects were allocated sequentially to the successive stages without randomisation.

*Stage I* consisted of harvesting the flap based on the superficial inferior epigastric vessels, in continuity with segments of the femoral vessels, obtaining an “inverted T” configuration (Figure 3(A1)). The flap was sutured back in its initial position through four end-to-end anastomoses, two arterial and two venous, between the femoral vessels and those of the flap (Figure 3(A2)).

*Stage II* involved mobilisation of both epigastric flaps, one of which was transferred to the contralateral inguinal region (Figure 3(B1)). The anastomoses performed were identical to those of the first stage (Figure 3(B2)), with the addition, in the donor area, of two end-to-end anastomoses (one arterial and one venous) restoring the blood flow of the corresponding hind limb.

In *Stage III*, the epigastric flap, mobilised in the same way as in the previous stages, was connected to the cervical vessels through three end-to-end anastomoses: two between the carotid artery and the epigastric artery harvested with parts of the femoral artery, and one between the external jugular vein and the epigastric vein in continuity with parts of the femoral vein. One end of the superficial inferior epigastric vein, as well as the distal end of the external jugular vein, were ligated. (Figure 3(C1,C2)).

*Stage IV* differed from the previous stage in that the epigastric flap was harvested with portions of the femoral vessel walls, as a patch, to provide an enlarged donor suturing interface and facilitating the anastomotic technique (Figure 3(D1)); the arterial anastomosis was performed in an end-to-side manner (Figure 3(D2)). This involved creating a breach in the wall of the carotid artery by placing a suture in the arterial wall, applying traction on it and excising the wall thus exposed with curved scissors.

In *Stage V*, the epigastric vessels were divided at their origin from the femoral vessels without retaining the patch (Figure 3(E1)), and the flap was transferred to the cervical region in the same manner as in the previous stage, namely an end-to-side anastomosis between the epigastric and carotid arteries and an end-to-end anastomosis between the epigastric and external jugular veins (Figure 3(E2)). The distinction therefore concerned the preparation of the donor anastomotic interface. Vessel diameters and mismatch ratios were not recorded and cannot be reported from the available data.

The results were assessed on the basis of outcome measures, applied uniformly across all stages: patency of the anastomosis both immediately and after 10 min; the character of minimal, moderate or severe blood leakage (recorded for patent anastomoses); and the time required to complete the anastomosis (duration of suture placement).

Leakage was assessed visually by the operating surgeon in patent anastomoses during the first 10 min after declamping. Minimal leakage ceased spontaneously without intervention by the operator, moderate leakage required temporary coverage with gauze or adjacent tissue and ceased within the 10 min assessment period, without meeting any criterion for severe leakage and severe leakage was defined by any of the following: bleeding that persisted at the end of the 10 min assessment despite intervention; leakage that flooded the operative field; or leakage requiring additional sutures to achieve haemostasis. The requirement for additional sutures constituted severe leakage even when bleeding subsequently ceased within the assessment period.

After completion of the final in vivo assessments in each animal, all anastomoses were excised and examined microscopically under continued anaesthesia, before euthanasia. Microscopic examination corroborated non-patency in anastomoses already classified as non-patent in vivo and did not alter the recorded patency classifications. Euthanasia followed completion of these assessments, as described above. All procedures were terminal, and no flap viability or survival outcomes were collected.

Procedural success required immediate patency with no or minimal leakage during the assessment period. Moderate or severe leakage constituted failure of the composite endpoint.

Given the exploratory, pilot character of the study, no a priori sample size calculation was performed. The number of animals allocated to each stage was the minimum judged sufficient to complete the operative protocol of that stage, in accordance with the principle of reduction. Statistical analyses were conducted with the aid of IBM SPSS Statistics, version 29.0.2.0.

Categorical outcomes (immediate patency, patency at 10 min and procedural success) are reported as proportions with 95% confidence intervals calculated by the Wilson score method. Comparisons between arterial and venous anastomoses, between end-to-end and end-to-side arterial configurations, and between inguinal and cervical recipient sites were performed with Fisher’s exact test; effect sizes are expressed as odds ratios with 95% confidence intervals. The presence of a monotonic trend in success and in 10 min patency across the five stages was tested with the Cochran–Armitage test for trend, stage number being entered as the ordinal score. Anastomosis time was summarised as mean ± standard deviation (SD) for each stage and vessel type. Separate ordinary least-squares linear regressions were fitted to the individual arterial and venous anastomosis times, with stage entered as an ordinal numerical predictor coded from 1 to 5. All recorded times were included, irrespective of patency or procedural success. Slopes are reported in minutes per stage with nominal 95% confidence intervals calculated using HC3 heteroscedasticity-consistent standard errors and t critical values with *n* − 2 degrees of freedom. Residual-versus-fitted and normal Q–Q plots were examined. Because within-animal dependence was not modelled, regression confidence intervals remain unadjusted for clustering and should be interpreted as exploratory. An illustrative sample-size calculation for future studies was performed using the normal approximation for two independent proportions, targeting 80% power. This calculation assumed independent observations and did not account for within-animal clustering or pairing.

All tests were two-sided and a *p*-value below 0.05 was considered to indicate statistical significance. No adjustment was made for multiplicity: apart from the comparison of arterial with venous anastomoses, all analyses are exploratory and are reported as such. Confidence intervals rather than *p*-values are emphasised throughout, since at the available sample size the precision of each estimate is considerably more informative than the outcome of a hypothesis test.

The study included 20 animals contributing 56 anastomoses, with multiple observations clustered within individual animals. Arterial and venous outcomes were obtained from the same animals and therefore constituted within-animal comparisons. The Wilson confidence intervals, Fisher’s exact tests, Cochran–Armitage trend tests and regression confidence intervals did not account for this dependence. HC3 standard errors address heteroscedasticity but not within-animal correlation. These analyses are consequently reported as unadjusted for clustering and exploratory.

## 3. Results

A total of 56 microvascular anastomoses were performed, 30 arterial and 26 venous. The results obtained for each stage, treating arterial and venous anastomoses separately, are presented in Table 1.

In Stage I, six arterial and six venous anastomoses were performed, distributed among the three subjects included, with overall success rates of 83.3% and 66.7%, respectively. Patent anastomoses showed no blood leakage, and the mean execution time was 19 min for arteries and 20 min for veins.

Stage II included the same number of anastomoses as the preceding one; all arterial anastomoses were patent, with an execution time of 20 min, whereas the venous anastomoses showed a success rate of 50%, with a duration of 23 min.

In Stage III, six of eight arterial anastomoses were patent, with a mean duration of 22 min. Half of the four venous anastomoses met the success criteria, with a mean time of 24 min.

In Stage IV, four of five arterial anastomoses were patent, one of which showed significant blood leakage. Among the venous anastomoses, three of five were patent, without leakage. The success rate in this stage was therefore 60% for both types of anastomoses.

The last stage included five arterial and five venous anastomoses, with success rates of 80% and 60%, respectively. No anastomotic leakage was present, and the mean execution time was 27 min for arterial and 31 min for venous anastomoses.

Considering all stages together, the overall success rate was 80.0% (24/30) for arterial and 57.7% (15/26) for venous anastomoses, while patency at 10 min was 70.0% (21/30) and 46.2% (12/26), respectively. Across all stages, mean anastomosis time was 22.33 ± 3.41 min for arterial and 24.96 ± 4.39 min for venous anastomoses.

Pooled across the five stages, procedural success was achieved in 80.0% of arterial anastomoses (24/30; 95% CI 62.7–90.5) and in 57.7% of venous anastomoses (15/26; 95% CI 38.9–74.5). The absolute difference of 22.3 percentage points corresponded to an odds ratio of 2.93 (95% CI 0.90–9.60) and did not reach statistical significance (Fisher’s exact test, *p*-value is 0.087). Patency at 10 min was 70.0% (21/30; 95% CI 52.1–83.3) for arterial and 46.2% (12/26; 95% CI 28.8–64.5) for venous anastomoses (OR 2.72, 95% CI 0.91–8.16; *p*-value is 0.103).

Immediate patency was 83.3% for arterial (25/30; 95% CI 66.4–92.7) and 61.5% for venous anastomoses (16/26; 95% CI 42.5–77.6), an absolute difference of 21.8 percentage points (OR 3.12, 95% CI 0.90–10.84; *p*-value is 0.079). The arterial–venous gap was therefore of almost identical magnitude at all three endpoints: 21.8 percentage points immediately, 23.8 at 10 min and 22.3 for composite success. Among anastomoses patent immediately, 4 of 25 arterial (16.0%) and 4 of 16 venous (25.0%) had lost patency by the 10 min assessment, a difference well within sampling variation (*p*-value is 0.69).

Of the 17 anastomoses that failed the composite success criterion, 15 did so because patency was not achieved immediately and 2 (one arterial, one venous) because of moderate or severe leakage in an otherwise would-be-patent anastomosis.

Stage-specific estimates were imprecise, the 95% confidence intervals spanning 40 to 60 percentage points (arterial success: Stage I 83.3%, 95% CI 44–97; Stage II 100%, 95% CI 61–100; Stage III 75.0%, 95% CI 41–93; Stage IV 60.0%, 95% CI 23–88; Stage V 80.0%, 95% CI 38–96; venous success: 66.7%, 95% CI 30–90; 50.0%, 95% CI 19–81; 50.0%, 95% CI 15–85; 60.0%, 95% CI 23–88; and 60.0%, 95% CI 23–88, respectively). The Cochran–Armitage test identified no significant monotonic trend across stages for any outcome: immediate patency (arterial z = −0.54, *p*-value is 0.59; venous z = −0.32, *p*-value is 0.75), composite success (arterial z = −0.88, *p*-value is 0.38; venous z = −0.07, *p*-value is 0.94) or patency at 10 min (arterial z = 0.03, *p*-value is 0.98; venous z = 0.10, *p*-value is 0.92).

Arterial success was 85.0% (17/20; 95% CI 64–95) in the three stages employing an end-to-end configuration and 70.0% (7/10; 95% CI 40–89) in the two stages employing an end-to-side configuration (*p*-value is 0.37). By recipient site, arterial success was 11/12 in the inguinal stages and 13/18 in the cervical stages (*p*-value is 0.36), and venous success 7/12 and 8/14 respectively (*p*-value is 1.00).

**Figure 4:**
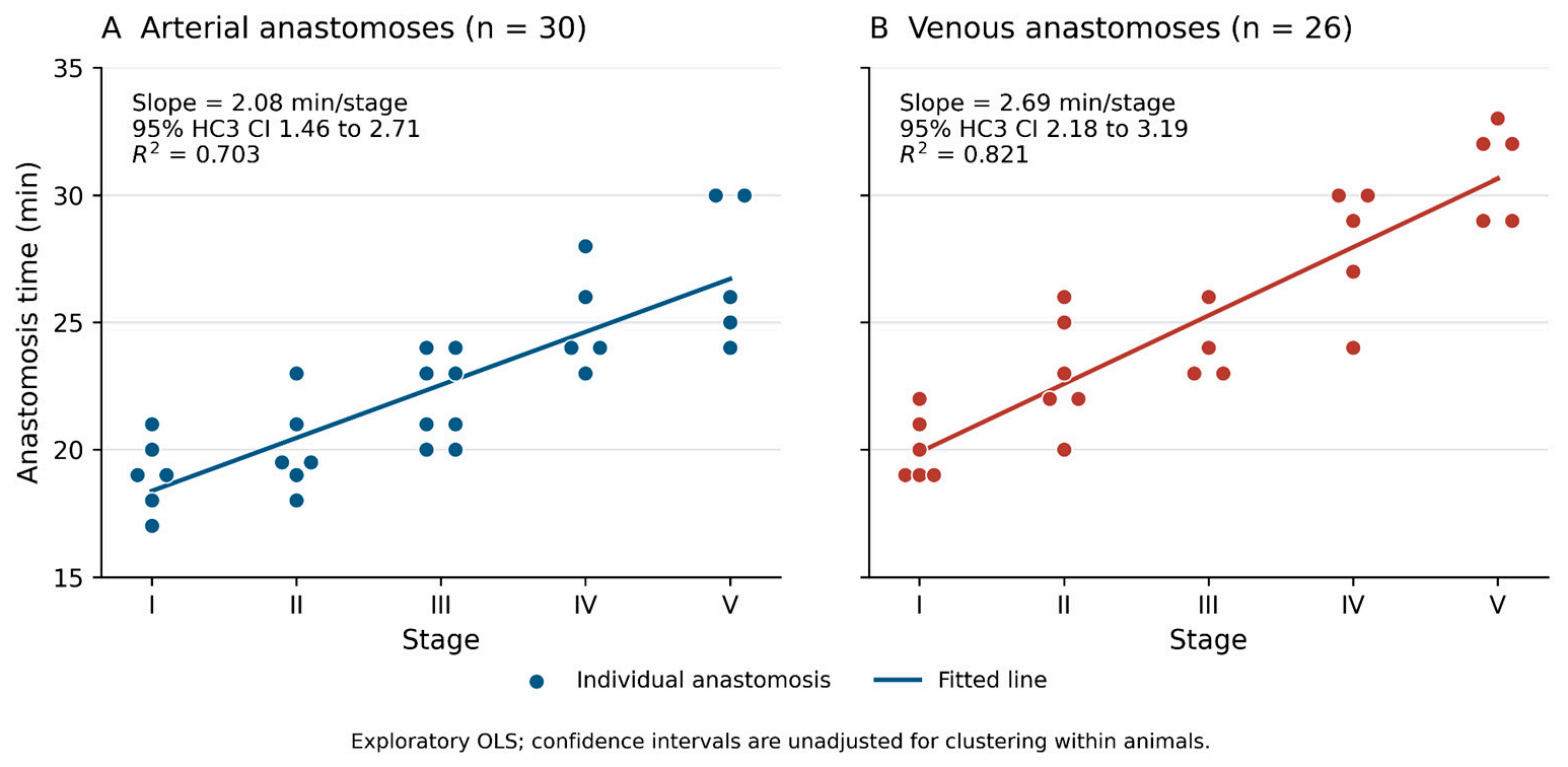
Individual anastomosis times across stages. Each point represents one arterial ((**A**); *n* = 30) or venous ((**B**); *n* = 26) anastomosis. Small horizontal offsets separate overlapping observations; recorded times and stage values used for regression were unchanged. Lines show separate ordinary least-squares fits with stage coded 1–5. Slope confidence intervals use HC3 standard errors and t critical values with *n* − 2 degrees of freedom and are unadjusted for within-animal clustering.

Anastomosis time increased with stage in the individual-level analyses (Figure 4). Among the 30 arterial anastomoses, the estimated slope was 2.08 min per stage (95% HC3 CI 1.46–2.71; R2 = 0.703). Among the 26 venous anastomoses, the estimated slope was 2.69 min per stage (95% HC3 CI 2.18–3.19; R2 = 0.821). Mean venous time exceeded mean arterial time at every stage, with differences of 1–4 min. Residual plots suggested greater arterial variability at later stages; the reported HC3 intervals allow for unequal variance. Residual-versus-fitted and normal Q–Q plots are presented in Figure S1. The regressions and their confidence intervals remain exploratory and unadjusted for within-animal clustering.

During the performance of the microvascular anastomoses, technical difficulties of variable frequency and intensity were identified, depending on the stage and on the type of anastomosis performed.

**Figure 5:**
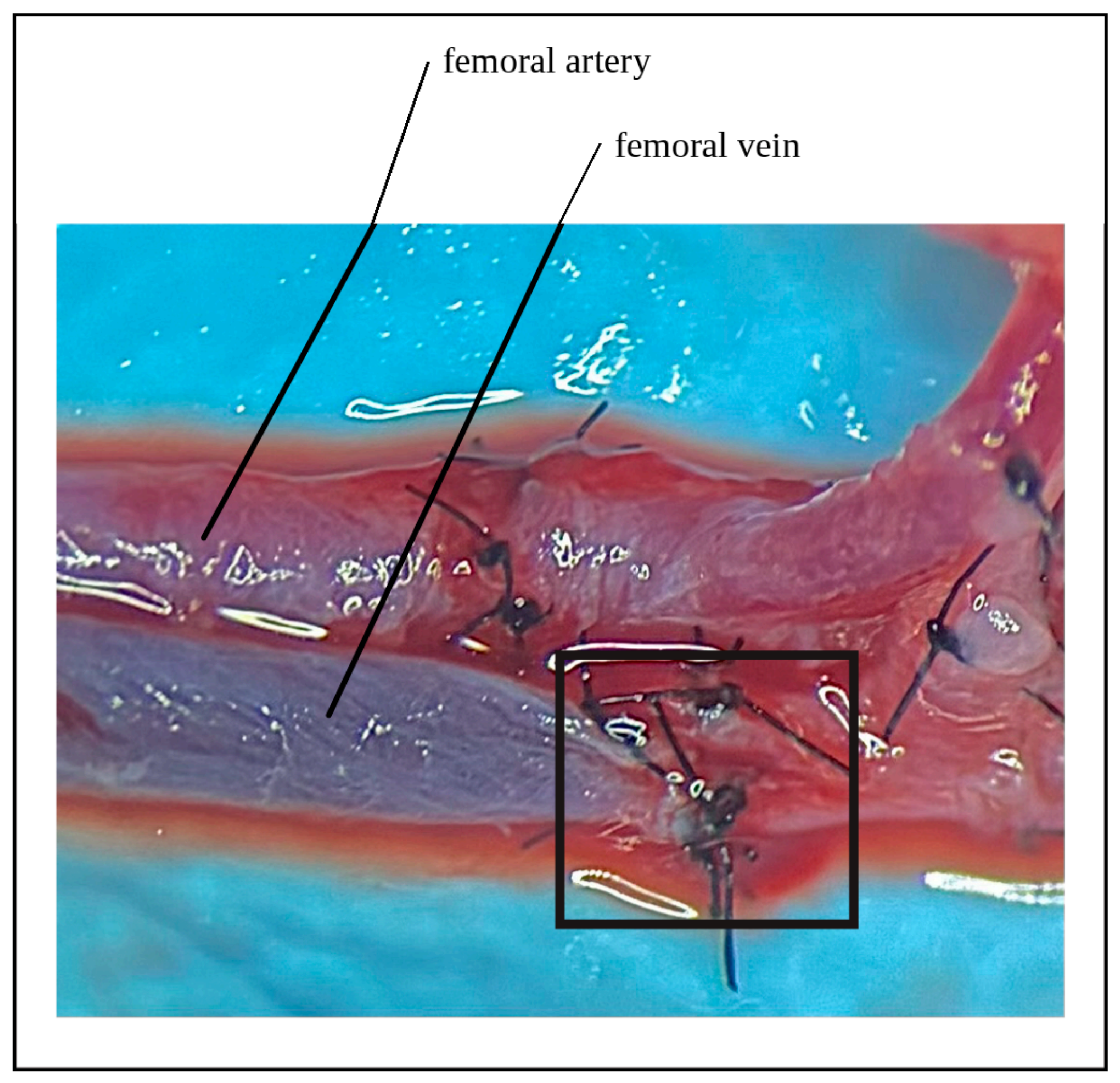
The frame contains a stenosing end-to-end venous anastomosis, caused by incorrect placement of the sutures simultaneously through both walls of the same blood vessel (microscopic image).

In the initial stages (I–II), difficulties were limited and consisted of the risk of catching the posterior wall due to lack of experience on living models (Figure 5).

**Figure 6:**
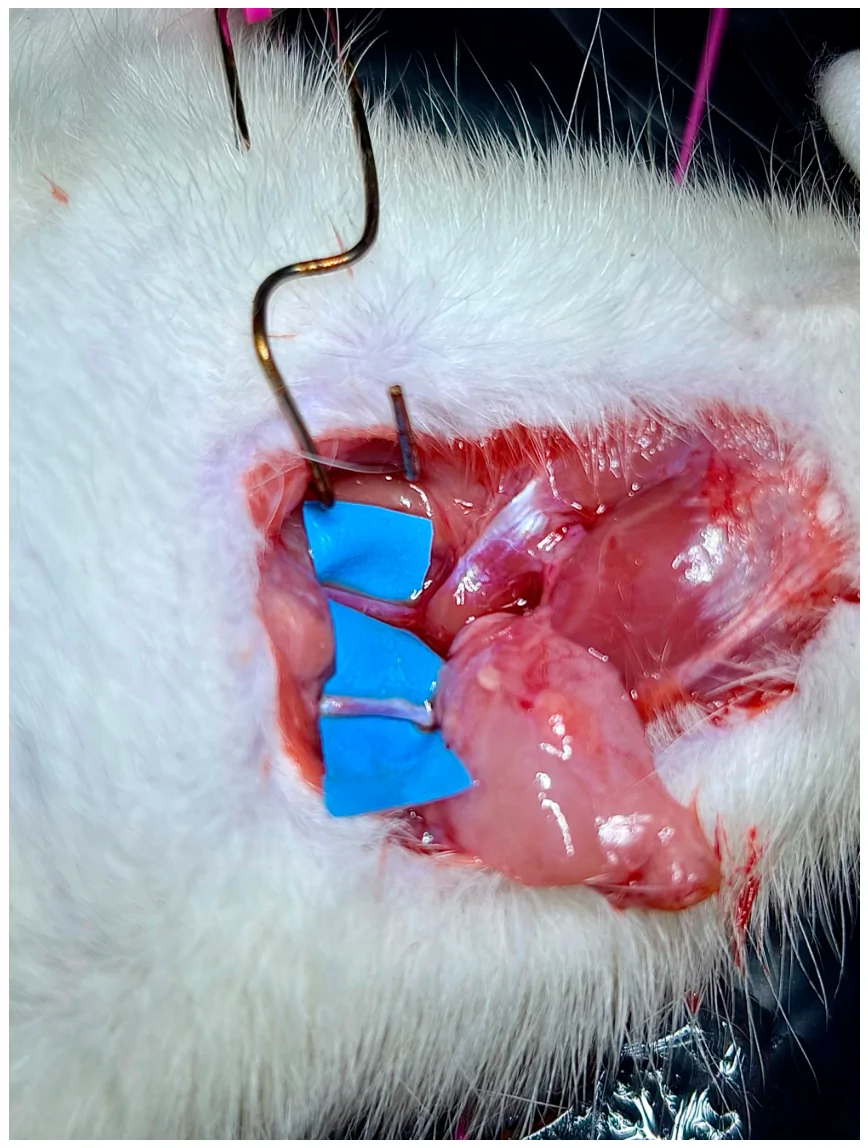
Blood vessels located in the depth of the cervical region.

The intermediate stage (III) added difficulty through a narrower working space (the cervical region), which contains anatomical elements that further limit movement in which the surgical technique needs to avoid resting instruments on the trachea or placing retractors across it under pressure. The blood vessels of the cervical region lie deeper than those of the inguinal region (Figure 6).

**Figure 7:**
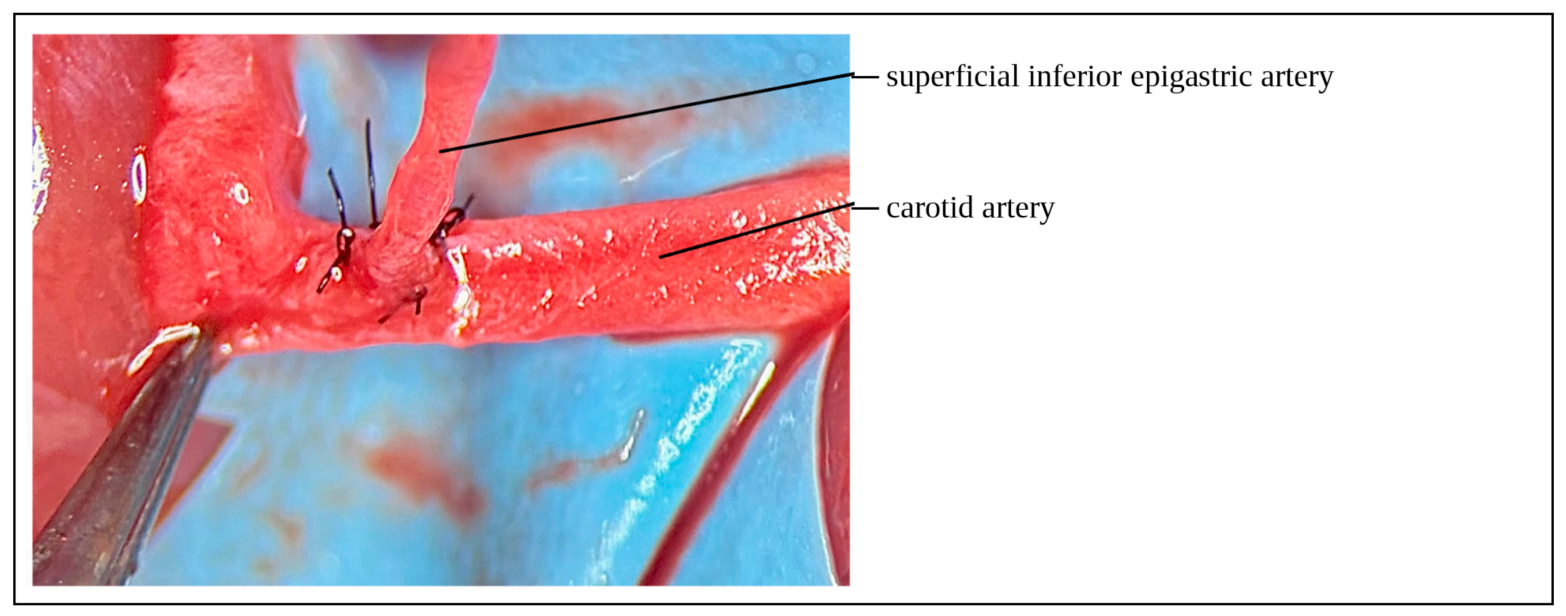
The calibre of the superficial inferior epigastric artery is significantly smaller than that of the carotid artery (microscopic image).

In the advanced stages (IV–V), technical difficulties were frequent, mainly related to the equidistant placement of suture points with minimal vessel injury, because of the reduced dimensions of the vessels (Figure 7).

**Figure 8:**
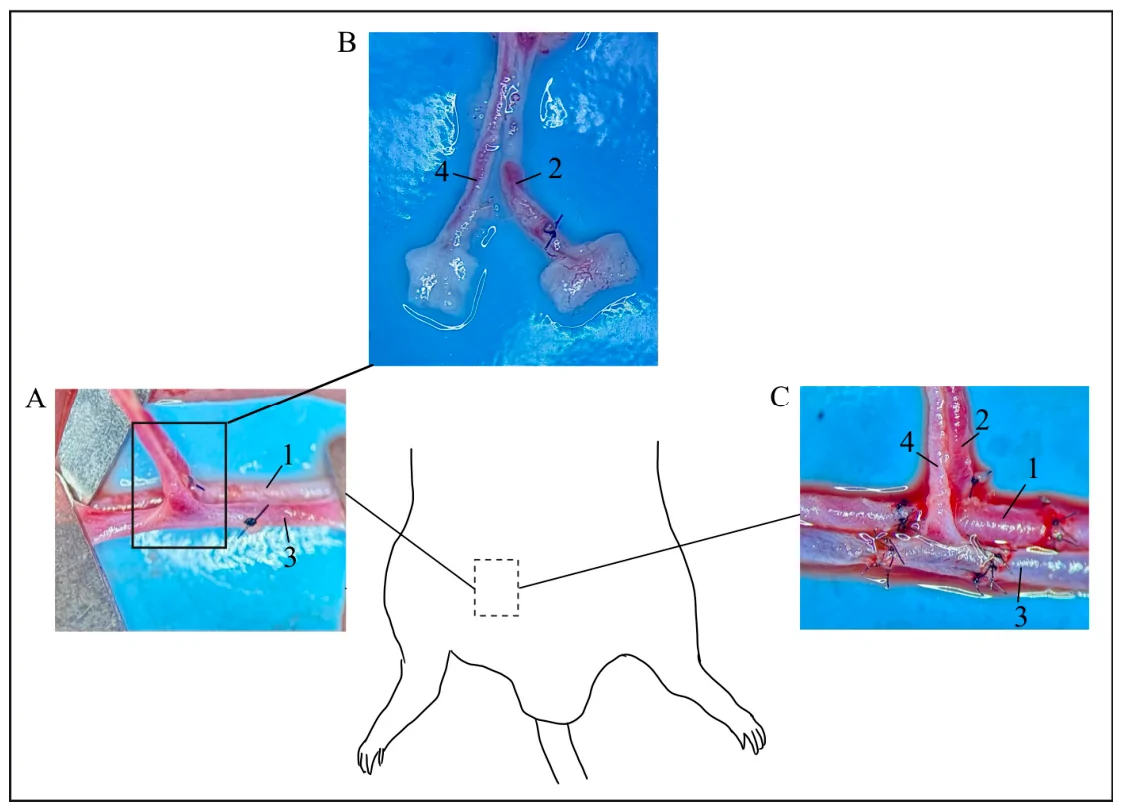
Microscopic images from Stage I. (**A**) Vascular arrangement of the inguinal region; (**B**) appearance of the epigastric vessels divided together with portions of the femoral vessels; (**C**) final appearance, after declamping, of the ipsilaterally performed anastomoses. 1 = femoral artery; 2 = superficial inferior epigastric artery; 3 = femoral vein; 4 = superficial inferior epigastric vein.

Representative technical aspects corresponding to the operative steps of the first stage are illustrated in Figure 8, showing blood flow present in both the artery and the vein after completion of the anastomoses and declamping (Figure 8C).

**Figure 9:**
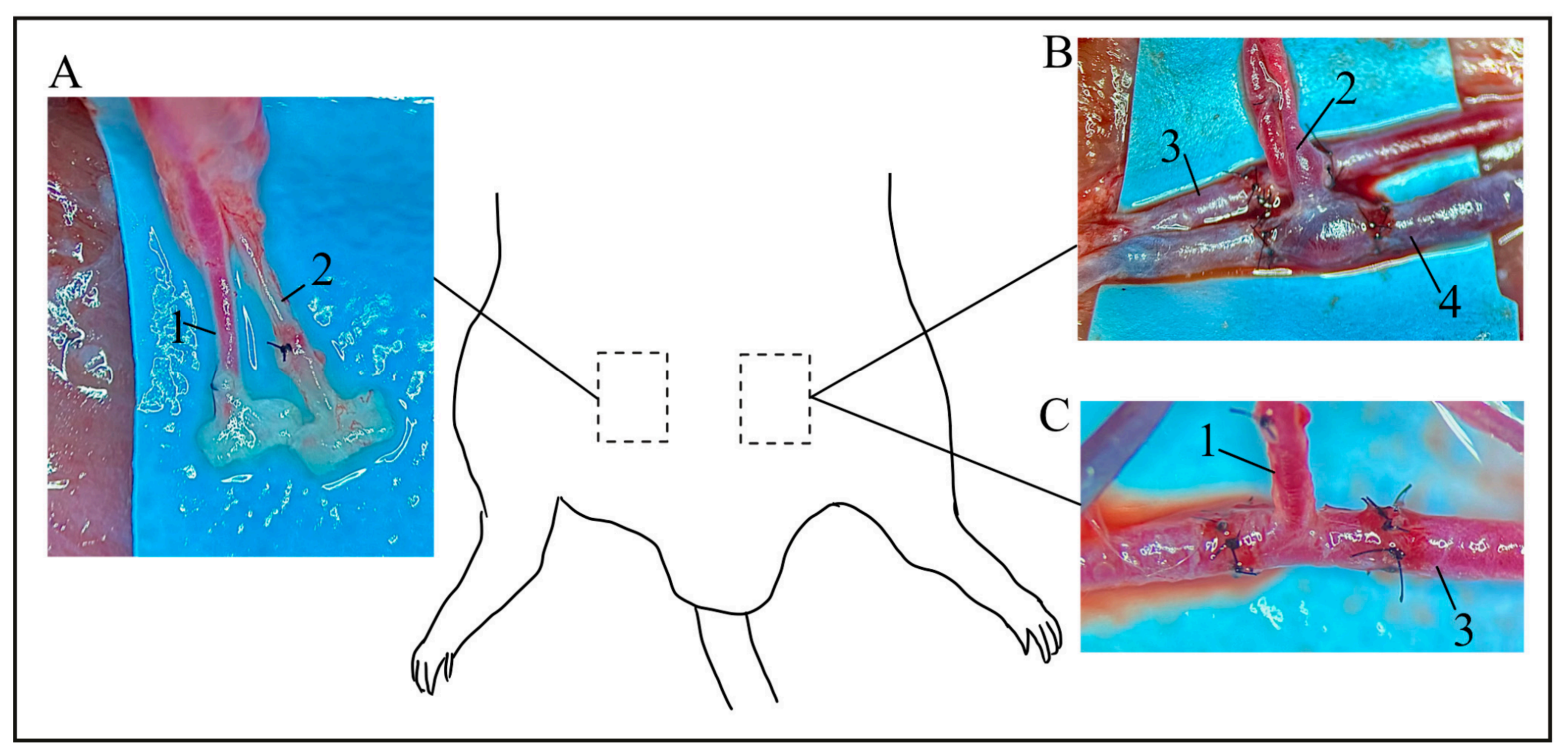
Microscopic images from Stage II. (**A**) Appearance of the epigastric vessels divided together with portions of the femoral vessels; (**B**) final appearance, after declamping, of the venous anastomoses performed in the contralateral inguinal region; (**C**) final appearance, after declamping, of the arterial anastomoses performed contralaterally. 1 = superficial inferior epigastric artery; 2 = superficial inferior epigastric vein; 3 = femoral artery; 4 = femoral vein.

Stage II involves approaching both inguinal regions, as shown in Figure 9. Correct alignment of the vessel wall edges and uniform distribution of the sutures can be observed for the arterial (Figure 9C) and venous (Figure 9B) anastomoses.

**Figure 10:**
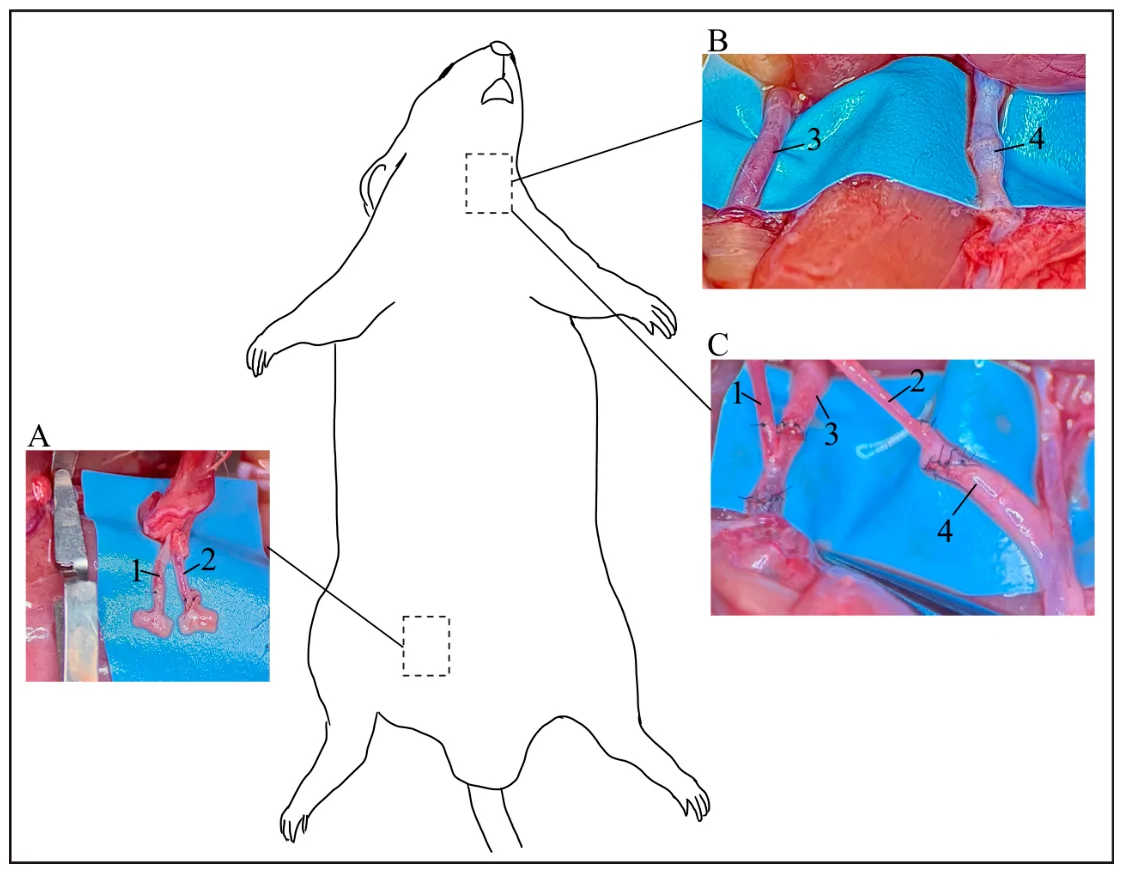
Microscopic images from Stage III. (**A**) Appearance of the epigastric vessels divided together with portions of the femoral vessels; (**B**) vascular arrangement of the cervical region; (**C**) final appearance, after declamping, of the arterial and venous anastomoses performed in the neck. 1 = superficial inferior epigastric artery; 2 = superficial inferior epigastric vein; 3 = carotid artery; 4 = external jugular vein.

The images from Stage III (Figure 10) reflect the deeper location of the cervical vessels and, consequently, of the working plane, compared with the inguinal region.

**Figure 11:**
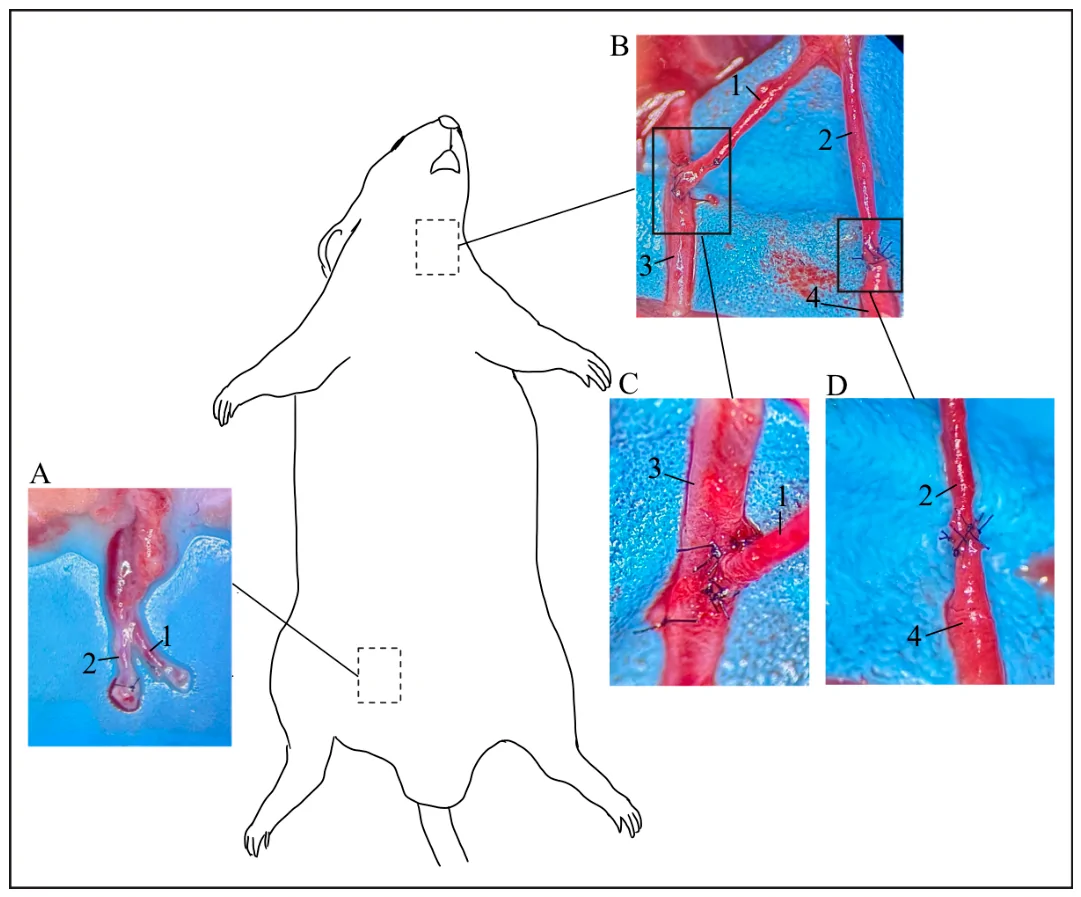
Microscopic images from Stage IV. (**A**) Appearance of the epigastric vessels divided together with limited portions of the femoral vessel walls, as a patch; (**B**) final appearance, after declamping, of the arterial and venous anastomoses performed in the neck; (**C**) end-to-side anastomosis between the carotid and epigastric arteries; (**D**) end-to-end anastomosis between the jugular and epigastric veins. 1 = superficial inferior epigastric artery; 2 = superficial inferior epigastric vein; 3 = carotid artery; 4 = external jugular vein.

From Stage IV onwards (Figure 11), the discrepancy between the stump diameter of the epigastric vessels and that of the carotid artery and external jugular vein, and their mismatch becomes evident. To accommodate this difference, the arterial anastomosis is performed in an end-to-side manner with the aid of the retained femoral vessel-wall patch to adjust for mismatch.

**Figure 12:**
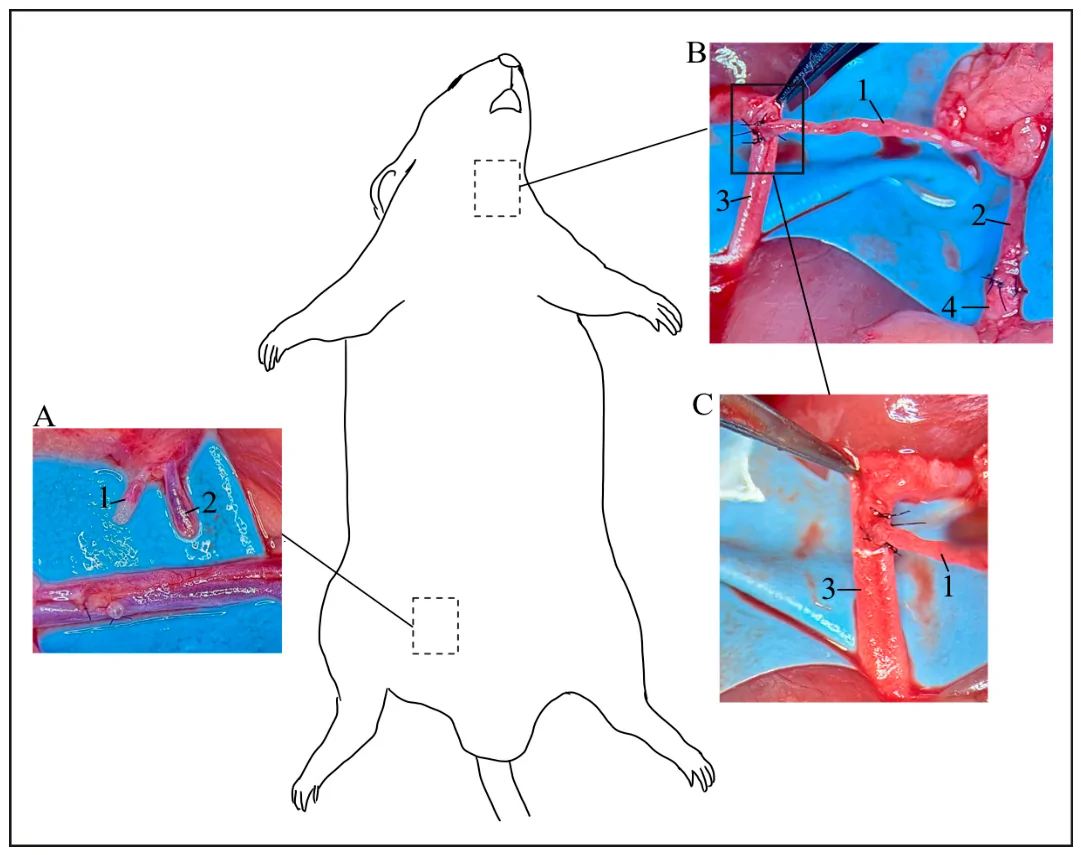
Microscopic images from Stage V. (**A**) Appearance of the epigastric vessels divided at their origin from the femoral vessels; (**B**) final appearance, after declamping, of the arterial and venous anastomoses performed in the neck; (**C**) end-to-side anastomosis between the carotid and epigastric arteries. 1 = superficial inferior epigastric artery; 2 = superficial inferior epigastric vein; 3 = carotid artery; 4 = external jugular vein.

Stage V presents the highest level of difficulty, because the epigastric vessels are harvested without portions of the femoral vessels (Figure 12A), which makes equidistant placement of the suture points more difficult during end-to-side anastomosis using the native epigastric arterial end (Figure 12B,C).

## 4. Discussion

This pilot study asked whether a single anatomical preparation, the rat superficial inferior epigastric flap, can carry a graded curriculum that takes a microsurgeon in training from non-living simulation to the threshold of clinical free tissue transfer, and whether the performance data such a curriculum generates are interpretable. Three findings emerged, each with a different implication. First, individual anastomosis time showed a positive association with stage for both vessel types, although fixed stage order prevented the effects of technical demand and accumulating experience from being separated. Second, procedural success did not decline significantly across stages; the stage-to-stage fluctuations observed are indistinguishable from sampling variation at this sample size, and we do not interpret them as a learning pattern. Third, the disadvantage of venous over arterial anastomoses was of almost identical magnitude at every endpoint and was already fully established at the moment of declamping, which places it in the act of suturing rather than in the subsequent behaviour of the anastomosis under flow. The curriculum is the contribution, and it is considered first; the three findings are then discussed in turn, followed by the design feature that generates the most demanding stages, by the position of the model within the existing literature, and by the constraints that limit what a study of this size can establish.

Before considering what the outcome data show, it is worth stating plainly what the study is for. The object of this work is a curriculum, not a dataset; the quantitative apparatus that follows exists to characterise that curriculum and to give a supervisor an objective reading of where a trainee stands within it. The problem addressed is a specific and well-recognised discontinuity in surgical education. A trainee who has completed a conventional non-living programme can suture a silicone tube or a chicken vessel competently [11,12,39], yet is not thereby prepared to perform a free tissue transfer. What is missing is not suturing. It is flap harvest, the management of a pedicle of finite length and fixed orientation, the conduct of two operative fields within one procedure, the handling of a calibre discrepancy that cannot be avoided and, above all, the experience of doing all of this where the vessel carries blood and where a technical error produces a visible and irreversible consequence. The live model is the only setting in which these demands can be met [25], and the function of staging is to present them one at a time rather than all at once.

The stages were designed to introduce additional technical demands while building on previously practised skills. Stage I introduces flap harvest and the reality of flow, but returns the flap to its own bed, so that vessel geometry, orientation and length remain those the trainee has just dissected; with four anastomoses per animal it is also the highest-yield stage for repetition. Stage II adds transfer itself: the pedicle must reach a new position, and the trainee meets for the first time the errors that transfer generates, namely torsion, kinking and insufficient length, while being required simultaneously to restore the circulation of the donor limb through a second and independent set of anastomoses. Stage III adds a second operative field, and with it recipient-site planning, a narrower and deeper working space, altered instrument angles and retraction that must not compromise the airway; this is the point at which the exercise begins to reproduce the ergonomics of head and neck reconstruction. Stage IV introduces an end-to-side arterial configuration while retaining a femoral-wall patch at the donor suturing interface so that the end-to-side technique can be learned using the enlarged donor suturing interface provided by the patch. Stage V uses the native epigastric vessel ends without this patch, requiring direct manipulation and suturing of these ends. This transition was intended to increase the demands of vessel handling and suture placement; changes in effective anastomotic dimensions and donor–recipient mismatch were not quantified.

The present pilot study characterised the operative stages and their immediate technical outcomes but did not validate competence-based progression thresholds. To support future educational implementation, Table 2 specifies the learning objectives, operative configurations, target competencies, assessment endpoints and proposed criteria for advancement. These criteria are intended for prospective evaluation and are distinct from the procedural-success endpoint analysed in the present study.

For future implementation, a stage would be passed after two consecutive successful complete stage exercises, each performed in a separate animal under supervision. During each exercise, every required arterial and venous anastomosis must be patent immediately after declamping and at the 10 min assessment, with no persistent suture-line bleeding at 10 min and no rescue suturing or anastomotic revision after initial declamping. All stage-specific competencies must be demonstrated without corrective prompting or hands-on technical assistance.

The supervising assessor would record each competency as demonstrated independently, demonstrated with assistance, or not demonstrated. Progression would require an independent rating for every competency and the absence of critical technical errors, including posterior-wall capture, vessel avulsion, or pedicle torsion or kinking that compromises flow. An unsuccessful assessment would interrupt the consecutive-success sequence and prompt targeted remediation, preferably on non-living models, before reassessment.

Anastomosis time would be recorded individually as a secondary measure of efficiency. No numerical time threshold is proposed because the present data do not establish a validated time-based standard. The two-exercise requirement is a provisional educational benchmark selected to require repeated successful performance; its reliability and predictive value require prospective validation. Completion of Stage V would indicate completion of this experimental module and would not, by itself, establish competence for independent clinical microsurgery.

The staged structure may help supervisors organise feedback around the principal technical demands emphasised at each level. Successful completion of preceding stages provides evidence of prior performance, but failure at a subsequent stage cannot be attributed exclusively to its newly emphasised demand. Basic technical errors, anatomical variation and physiological factors may also contribute. The proposed progression criteria therefore combine anastomotic outcomes with direct assessment of operative technique, allowing observed errors to guide targeted remediation. The reliability of this approach and its ability to identify specific training needs require prospective evaluation in a cohort of trainees.

Completion of Stage V should provide a basis for considering supervised clinical participation, with readiness assessed through both anastomotic outcomes and operative technique. We propose that trainees demonstrate reproducible arterial and venous patency immediately after clamp release and at the 10 min assessment, absence of persistent anastomotic leakage, atraumatic tissue handling, accurate suture placement, secure knot tying and appropriate vessel alignment across repeated assessments. Critical errors, including posterior-wall capture and flow-compromising tension or twisting, should prompt targeted remediation and reassessment before progression. Technical performance could be evaluated by an experienced microsurgeon using the University of Western Ontario Microsurgical Skills Acquisition/Assessment (UWOMSA) instrument, which has demonstrated reliability and validity in laboratory and clinical assessments [40,41]. Anastomosis time should remain a supplementary measure of efficiency, interpreted alongside technical quality.

Transition to clinical microsurgery should involve graduated participation in appropriately selected cases under direct supervision, beginning with individual operative steps and progressing to complete anastomoses as competence is demonstrated. The supervising microsurgeon should determine the level of responsibility according to the trainee’s observed performance and the complexity of the case. Clinical progression must additionally address surgical judgement, recognition and management of anastomotic failure, and the anatomical and pathological variability of human vessels. The present study did not evaluate trainee progression or subsequent clinical performance and therefore cannot establish a validated assessment score, number of successful anastomoses or time threshold for clinical readiness. These proposed transition criteria require prospective evaluation before they can be adopted as formal proficiency standards.

Because each stage retains the demands of all preceding ones and adds a single new demand, a trainee who has completed Stage III and then fails at Stage IV has demonstrated a deficit localised to the management of calibre mismatch, and not to dissection, exposure, vessel handling or suture technique, all of which the preceding stage has already established. Curricula that advance by changing the substrate cannot offer this, because a decline in performance following a transition from synthetic material to living tissue is compatible with several unrelated explanations.

Two further considerations follow from building the ladder in this way. The first concerns the responsible use of animals. The stages are arranged so that most anastomotic repetitions occur early, in the technically simpler configurations, and the live subject is committed to the advanced configurations only where no non-living substitute exists; twenty animals yielded fifty-six anastomoses, three orthotopic replantations and seventeen flap transfers, the preparatory competences having been acquired beforehand on non-living material [11]. The second concerns where the curriculum ends. Stage V is not a terminal qualification but the point at which the trainee has met, in a controlled experimental setting, each of the demands that a clinical free flap will impose. What the model offers at that point is a reasoned basis for supervised progression to the operating theatre, which is the transition the whole structure exists to make safer [10,24].

Mean anastomosis time increased from 19.00 ± 1.41 to 27.00 ± 2.83 min for arteries and from 20.00 ± 1.26 to 31.00 ± 1.87 min for veins between Stages I and V. Regression of individual times estimated increases of 2.08 min per stage for arteries and 2.69 min per stage for veins. The corresponding R2 values of 0.703 and 0.821 indicate a substantial association with stage while retaining the variability between individual anastomoses. The data therefore support an increasing time requirement across the proposed stages, without establishing a deterministic relationship or equal increments in difficulty.

The single operator completed the stages in a fixed ascending sequence, so stage and cumulative experience advanced together. The longer times at later stages are consistent with the intended increase in technical demand, but the effects of task difficulty and accumulating experience cannot be separated in this design. These results characterise performance within this pilot series and do not establish a learning curve, a causal effect of stage, or a validated measure of competence. Confirmation requires a larger study with multiple operators and a design that separates stage from practice order.

With four to eight anastomoses per stage and vessel type, the 95% confidence interval around any single stage estimate spans 40 to 60 percentage points, and every interval overlaps every other. Formal testing for a monotonic trend across stages found none, for success (arterial *p* = 0.38; venous *p* = 0.94) or for 10 min patency (*p* = 0.98 and *p* = 0.92 respectively). The apparent maxima in Stage II and Stage V correspond to a difference in one or two anastomoses and would be reversed by a single additional failure. We therefore make no claim of a two-phase learning pattern in these data. The defensible statement is narrower but not trivial: the success rate was broadly maintained while task difficulty increased and operative time lengthened, which is consistent with, though not proof of, a compensatory gain in skill.

This carries a concrete design implication for anyone extending the model. A study powered to detect a 20-percentage-point difference in success rate at conventional thresholds would require approximately 66 anastomoses per group. Questions about learning in microsurgery are consequently better addressed either with substantially larger series, or with continuous endpoints such as operative time, or with sequential monitoring methods designed to detect performance shifts within small samples. Reporting stage-wise proportions without their confidence intervals, as is common in the experimental training literature, invites precisely the over-interpretation avoided here, and is one of the practices that standardisation initiatives in experimental microsurgery have sought to correct [42].

Arterial anastomoses outperformed venous ones on every endpoint, and by a remarkably constant margin: immediate patency 83.3% (25/30) against 61.5% (16/26), a difference of 21.8 percentage points; patency at 10 min 70.0% against 46.2%, a difference of 23.8 points; and composite success 80.0% against 57.7%, a difference of 22.3 points. None of the three comparisons reached conventional significance (*p* = 0.079, 0.103 and 0.087 respectively), and each confidence interval remains compatible both with a negligible difference and with a threefold odds ratio. The consistency of the gap across three endpoints that fail for different reasons nevertheless makes it difficult to attribute wholly to chance, and the more instructive question is not whether it exists but when it arises.

The gap was already fully present at the moment of declamping and did not widen thereafter. Among anastomoses patent immediately, 4 of 25 arterial (16.0%) and 4 of 16 venous (25.0%) had lost patency at 10 min, a difference well within sampling variation (*p* = 0.69). The venous deficit was therefore created during suturing and subsequently maintained, rather than generated by the behaviour of the completed anastomosis under flow.

This localises the dominant mechanism to technical execution and corresponds closely to the established taxonomy of intraoperative microsurgical error [43]. The two errors most frequently identified in that taxonomy, catching the posterior wall while suturing the anterior one and incomplete penetration of the wall with the needle passing only through the adventitia, are both far more probable in a vein than in an artery. A thin, low-turgor, collapsible wall does not hold its lumen open once the vessel is emptied between the clamps, so the two walls lie in apposition at precisely the moment the needle is passed; an elastic arterial wall retains its shape and separates them. The corollary for training is specific rather than general: the venous limb of this model rewards an identifiable set of manoeuvres, namely adequate dilatation before suturing, deliberate presentation of the anterior wall away from the posterior one, and grasping the needle at a distance from its base so that the angle of entry remains oblique. It does not simply reward more repetitions of the same gesture, and a curriculum that responds to poor venous results with additional undifferentiated practice is unlikely to be efficient.

It would be wrong to conclude from the absence of an excess venous attrition within ten minutes that thrombosis is unimportant in this model. Early thrombosis of the rat femoral vein after end-to-end anastomosis is well documented, occurs irrespective of the immediate technical result, and has been attributed to dehydration of the animal with consequent hypotension and to cooling of the operative field with peripheral vasoconstriction [44]. Such events develop over an interval considerably longer than the one observed here. A ten-minute window tests the immediate mechanical result; it does not test thrombotic risk. The same applies to residual adventitia or intimal injury at the suture line, which exposes subendothelial collagen and tissue factor, initiates platelet adhesion through von Willebrand factor and activates the extrinsic pathway [37]; the consequences of such exposure fall largely outside the period we assessed, and are expected to be less well tolerated in a low-shear venous conduit than in an arterial one. The defensible statement is that within the period observed the model did not penalise veins selectively once flow had been restored, and that quantifying the thrombotic contribution requires a longer window.

One further observation follows from the decomposition and bears directly on the design of the endpoint. Of the 17 anastomoses that failed the composite success criterion, 15 failed because patency was not achieved immediately and only 2, one arterial and one venous, because of moderate or severe leakage in an otherwise patent anastomosis. The composite endpoint was therefore in practice almost identical to immediate patency: the leakage component contributed essentially no additional discrimination while introducing the only subjective judgement in the entire assessment. Either the leakage scale should be anchored to an explicit, pre-specified and independently verifiable definition, or it should be replaced by a measure that is genuinely orthogonal to patency, such as quantified anastomotic time under clamp or a structured rating of technique.

Stages IV and V used an end-to-side arterial configuration with different donor anastomotic interfaces. Arterial success was 85.0% across the three end-to-end stages and 70.0% across the two end-to-side stages (*p* = 0.37). Because donor preparation and operative setting also differed, this comparison does not isolate the effect of anastomotic configuration. Options for managing donor–recipient calibre mismatch include differential dilatation, oblique arteriotomy, interposition grafting and end-to-side anastomosis [45], the latter having been studied in rat arterial models [23,27]. Stage IV retained a femoral vessel-wall patch to enlarge the donor suturing interface, whereas Stage V used the native epigastric vessel ends without this patch. The Barcelona consensus describes vascular supermicrosurgery in relation to vessels of 0.3–0.8 mm [46], and published anatomical work supports the use of rat superficial epigastric vessels for supermicrosurgical training [47]. However, vessel-diameter measurements were not systematically recorded in the present study, preventing confirmation that Stage V met these diameter-based criteria. The distinction between Stages IV and V is therefore defined here by the preparation of the donor anastomotic interface.

The arterial success rates in Stages V and IV were 80.0% and 60.0%, respectively, but with five arterial anastomoses per stage, this difference represents only one successful anastomosis and does not establish that anastomosis without the femoral vessel-wall patch was easier.

Mean anastomosis time was higher in Stage V than in Stage IV, increasing from 25.00 ± 2.00 to 27.00 ± 2.83 min for arteries and from 28.00 ± 2.55 to 31.00 ± 1.87 min for veins. This descriptive difference is consistent with greater technical demand, although these small groups do not establish a separate effect of the Stage IV–V transition.

Non-living models are inexpensive, readily stored and free of ethical constraint, and they permit graded progression through tissues of differing wall thickness and flexibility, but they lack circulation and therefore cannot report patency, leakage or thrombosis [13,39]. Living models restore precisely these endpoints, allowing iatrogenic haemorrhage and its evolution to be observed and anastomotic patency to be verified over time, and they most closely reproduce the dissection, flap elevation and vessel handling of clinical microsurgery [25,26]. The rat epigastric flap occupies a well-established position within this second category: the dissection plane lies above the muscular fascia, donor-site morbidity is low, and an experienced surgeon transfers the flap to the cervical region with minimal or absent necrosis in 70 to 80% of cases [34]. Its similarity to clinical free tissue transfer accounts for its widespread use in reconstructive research [32,35] and for its recommendation as an entry model for perforator-based abdominal wall flaps [36].

Staged curricula have been described by several centres, most of them progressing across substrates, from synthetic tubes to non-living tissue and finally to the live animal [19,20,21,22,48]. The distinguishing feature of the present protocol, and the source of the diagnostic property aforementioned, is that progression occurs within a single anatomical model. The measurement consequence is worth stating separately from the educational one. When a curriculum advances from silicone to chicken wing to rat, each transition alters the tissue, the handling characteristics, the visual field and the feedback available simultaneously with the technical demand, so that a change in performance cannot be attributed to any one of them. Using a common flap model, operator, instrumentation and assessment method reduces several sources of variation between stages. Nevertheless, cumulative experience also changes with stage, so the time gradient cannot be attributed exclusively to task difficulty. Comparison between operators or centres would require prospective validation.

The endpoints adopted here, patency assessed at two time points, graded leakage and anastomosis time, are among those most frequently used to document microsurgical learning [49] and correspond to the parameters recommended for standardisation in experimental microsurgical training [42]. Their combination into a composite success criterion, defined a priori as immediate patency without moderate or severe leakage, is what permits the stage-by-stage comparison; their separate reporting is what permitted the failure-mode analysis, which in turn showed the composite to be largely redundant with immediate patency in this series. Both the composite and its components should therefore be reported, and the composite should not be reported alone.

The study has multiple limitations. The principal limitation is statistical. Twenty animals yielded 56 anastomoses distributed unevenly across ten stage-by-vessel groups containing between four and eight observations each, limiting precision, particularly for stage-specific binary outcomes. The venous cell of Stage III contained only four anastomoses, because the ligation of one epigastric venous end and of the distal external jugular vein permits a single venous anastomosis per subject in that configuration, while two arterial anastomoses are performed. Stages are therefore not equally precise, and the pooled arterial and venous totals are not balanced. Incomplete documentation prevented a comprehensive, stage-specific analysis of vessel diameters and donor–recipient calibre mismatch. The original microscopic image files and spatial calibration data were unavailable, preventing the reliable retrospective addition of scale bars. The photographs therefore provide qualitative illustrations of operative anatomy and anastomotic configuration and cannot substantiate absolute vessel dimensions.

The statistical analyses did not account for clustering of anastomoses within animals or for the within-animal pairing of arterial and venous outcomes. Although HC3 standard errors were used to accommodate unequal residual variance, they do not account for within-animal correlation. Positive correlation among observations from the same animal may lead to underestimated uncertainty and overly narrow confidence intervals. For arterial–venous comparisons, the effect of ignoring pairing depends on the correlation structure, which was not quantified. The analyses therefore remain exploratory, and the observed stage–time associations should be interpreted cautiously and confirmed in studies with appropriate adjustment for within-animal dependence.

The single-operator design has two consequences beyond those already discussed. It removes inter-operator variability, which is desirable for isolating the effect of stage, but it also means that the trajectory described is that of one individual with a defined prior level of non-living training, so the external validity of the absolute values to a cohort of trainees is unestablished. More fundamentally, because stage and experience advance together, the difficulty effect and the learning effect cannot be separately quantified. A design in which the stage order is varied between operators would resolve this.

Outcome assessment was performed by the operating surgeon without blinding. This is of limited concern for patency, which was verified both by the Acland test and by microscopic inspection of the excised anastomosis, but it is a material concern for the grading of leakage. Although operational descriptions of the leakage categories are provided, these were reconstructed retrospectively and were not based on a prospectively standardised or validated grading scale. Leakage classification depended on visual assessment and operator decisions regarding haemostatic intervention, introducing potential observer bias into the composite success endpoint. Since leakage enters the composite success criterion, a proportion of the difference between success and immediate patency, most visibly in Stage IV, rests on a subjective judgement. Independent assessment of video-recorded anastomoses against an explicit rating scale would remove this.

The observation window of 10 min is short relative to the clinically relevant period of thrombotic risk, and no flap survival data were collected. The study therefore reports an intraoperative surrogate rather than the outcome by which free tissue transfer is ultimately judged. This constraint bears directly on the interpretation offered in the prior discussion: the finding that veins did not lose patency disproportionately after declamping is bounded by the length of the window and cannot exclude a thrombotic excess emerging later, so it should be read as a statement about the immediate mechanical result rather than about thrombotic risk. Finally, no physiological monitoring was performed: arterial pressure, core and field temperature and haematocrit were not recorded. Future studies incorporating a survival model and extended postoperative monitoring are needed to assess sustained patency and flap survival.

Documentation of animal provenance was incomplete. Although the supplier identified the animals as Wistar rats, formal documentation confirming their stock identity and a more specific stock designation were unavailable. This limits independent verification of the animals’ stock identity and the completeness of provenance reporting.

This study may have implications of development. The future priority would be a series adequately powered for its principal comparison, of the order of 66 anastomoses per group, ideally distributed across several operators of comparable baseline so that individual trajectories can be separated from the stage effect. Video recording with independent, blinded assessment against a structured rating scale would address both the leakage grading and the absence of a validated global measure of technique. Extending the observation window to flap survival at 24 h and 7 days would convert the intraoperative surrogate into an outcome, and the concurrent recording of arterial pressure, temperature and haematocrit would allow the physiological hypothesis for venous failure to be tested rather than inferred.

The difficulty ladder itself admits further extension: a venous end-to-side configuration, greater deliberate calibre discrepancy, and recruitment of other vessels of the rat may be used. Because the substrate is fixed, such stages would remain commensurable with the existing ones. If the time-per-stage relationship reported here proves reproducible in larger and multi-operator series, it would provide the quantitative scale against which individual progression, and ultimately readiness for supervised clinical microsurgery, could be benchmarked between training centres.

## 5. Conclusions

The contribution of this work is a five-stage preclinical training pathway based on the rat superficial inferior epigastric flap. The stages introduce flap harvest under blood flow, transfer of a pedicle of finite length, a second operative field, an end-to-side arterial configuration with a retained femoral vessel-wall patch, and finally direct handling and suturing of the native epigastric vessel ends without this patch. The quantitative analysis characterises performance across these stages.

The clearest descriptive finding was the positive association between stage and individual anastomosis time, with estimated increases of 2.08 min per stage for arteries and 2.69 min per stage for veins. This is consistent with the intended increase in technical demand, but fixed stage order and unmodelled within-animal dependence limit interpretation. Anastomosis time appears useful for characterising the proposed stages, while its ability to measure learning or guide progression requires validation.

Procedural success, by contrast, showed no significant trend across stages (*p* = 0.38 for arterial and *p* = 0.94 for venous anastomoses), and the stage-to-stage fluctuations observed fall within sampling variation; we therefore report that success was maintained as difficulty increased, rather than claiming a demonstrated learning curve.

Venous anastomoses performed less well than arterial ones on every endpoint, by a nearly constant margin of approximately 22 percentage points (composite success 57.7% versus 80.0%). None of these differences reached significance at this sample size, but their distribution in time is informative: The arterial–venous difference was already evident at declamping (immediate patency 61.5% versus 83.3%), suggesting that technical factors may have contributed. However, neither this timing nor the subsequent 10 min assessment (25.0% versus 16.0%, *p*-value is 0.69) establishes the mechanism of failure or excludes early thrombosis.

The thin, collapsible venous wall can make separation of the anterior and posterior walls difficult during needle passage, increasing the risk of posterior-wall capture. Other potential errors include incomplete wall penetration, excessive or uneven suture bites, inversion of the vessel edges, and excessive knot tension. These may impair intimal apposition or distort the lumen, while excessive traction can damage the vessel wall. Training should therefore emphasise clear visualisation of the lumen, atraumatic handling, controlled full-thickness needle passage, evenly spaced sutures, accurate intima-to-intima approximation and knot tightening without luminal constriction. Although these mechanisms provide plausible explanations for poorer venous performance, their individual contributions were not systematically quantified in this study.

A methodological finding of practical consequence is that the composite success criterion proved almost redundant with immediate patency: only 2 of 17 failures were attributable to leakage in an otherwise patent anastomosis. The leakage component added the only subjective judgement in the assessment without adding discrimination, and should be either anchored to an explicit definition or replaced.

These conclusions are constrained by a small and unevenly distributed sample, a single unblinded operator, a semi-quantitative leakage criterion, a ten-minute observation window and the absence of physiological monitoring; all analyses are exploratory. Within these limitations, the model provides a standardised framework for preclinical free flap training, with quantifiable measures of anastomotic performance. The lower observed venous patency and procedural success rates suggest that venous anastomosis warrants particular attention in training and further investigation, although these differences were not statistically significant in this exploratory series. Confirmation in a larger, multi-operator series with blinded assessment and flap survival endpoints is the necessary next step before the protocol can be recommended for formal incorporation into residency curricula.

## Figures and Tables

**Table 1:** Results obtained in each stage, with arterial and venous anastomoses treated separately. Proportions are reported with nominal 95% Wilson confidence intervals. Percentages and confidence limits are rounded to one decimal place. Confidence intervals are calculated at the anastomosis level without adjustment for within-animal clustering and should be interpreted as exploratory. Anastomosis times are reported as mean ± SD. Anast. = anastomosis; EE = end-to-end; ES = end-to-side; A = arterial; V = venous; *n* = number of anastomoses.

Stage	Anast. Type	No. of Subjects	No. of Anast. Performed	Immediate Patency, % (95% CI)	Patency at 10 min, % (95% CI)	Patent, with No Leakage/Minimal Leakage (*n*)	Procedural Success, % (95% CI)	Anastomosis Time, Mean ± SD (min)
I	EE A	3	6	83.3 (43.6–97.0)	66.7 (30.0–90.3)	5	83.3 (43.6–97.0)	19.00 ± 1.41
I	EE V	3	6	66.7 (30.0–90.3)	33.3 (9.7–70.0)	4	66.7 (30.0–90.3)	20.00 ± 1.26
II	EE A	3	6	100.0 (61.0–100.0)	66.7 (30.0–90.3)	6	100.0 (61.0–100.0)	20.00 ± 1.41
II	EE V	3	6	66.7 (30.0–90.3)	66.7 (30.0–90.3)	3	50.0 (18.8–81.2)	23.00 ± 1.76
III	EE A	4	8	75.0 (40.9–92.9)	75.0 (40.9–92.9)	6	75.0 (40.9–92.9)	22.00 ± 2.19
III	EE V	4	4	50.0 (15.0–85.0)	25.0 (4.6–69.9)	2	50.0 (15.0–85.0)	24.00 ± 1.69
IV	ES A	5	5	80.0 (37.6–96.4)	80.0 (37.6–96.4)	3	60.0 (23.1–88.2)	25.00 ± 1.41
IV	EE V	5	5	60.0 (23.1–88.2)	60.0 (23.1–88.2)	3	60.0 (23.1–88.2)	28.00 ± 2.00
V	ES A	5	5	80.0 (37.6–96.4)	60.0 (23.1–88.2)	4	80.0 (37.6–96.4)	27.00 ± 2.83
V	EE V	5	5	60.0 (23.1–88.2)	40.0 (11.8–76.9)	3	60.0 (23.1–88.2)	31.00 ± 1.87

**Table 2:** Proposed educational objectives, assessment endpoints and progression criteria for the five-stage microsurgical training programme. SIE, superficial inferior epigastric; EE, end-to-end; ES, end-to-side. Common pass criteria are defined below. All progression criteria are proposed and require prospective validation.

Stage	Learning Objectives	Vessels and Configurations	Anastomoses per Animal	Target Competencies	Assessment Endpoints	Proposed Progression Criterion
I: Orthotopic replantation	Integrate flap harvest with anastomosis under circulating blood flow	SIE flap retained in continuity with femoral vessel segments; femoral arterial and venous EE anastomoses	4: 2 arterial 2 venous	Atraumatic dissection; preservation of pedicle orientation; vessel preparation; accurate suture placement and knot tension	Immediate and 10 min patency; leakage; technical checklist; individual anastomosis times	Two consecutive complete exercises meeting all common pass criteria before advancement to Stage II
II: Contralateral inguinal transfer	Transfer the flap while maintaining suitable pedicle length and orientation	Donor femoral vessel segments connected to contralateral femoral vessels by EE anastomoses; donor-limb femoral continuity restored by additional EE repairs	6: *4 flap anastomoses 2 donor-limb repairs* 3 arterial 3 venous	Recipient-site preparation; tension-free positioning; avoidance of pedicle torsion and kinking; restoration of donor-limb vascular continuity	Common endpoints, plus pedicle alignment and patency of donor-limb repairs	Two consecutive complete exercises meeting all common pass criteria, including donor-limb reconstruction, before advancement to Stage III
III: Cervical transfer with retained femoral segments	Perform transfer and anastomosis in a second, deeper operative field	Femoral arterial segment retained with the flap connected to the common carotid artery by two EE anastomoses; retained femoral venous segment connected to the external jugular vein by one EE anastomosis	3: 2 arterial 1 venous	Cervical recipient-vessel exposure; appropriate retraction; instrument control at depth; tension-free pedicle positioning	Common endpoints, plus assessment of cervical exposure and pedicle geometry	Two consecutive complete exercises meeting all common pass criteria before advancement to Stage IV
IV: Cervical transfer with femoral-wall patches	Perform arterial ES anastomosis using an enlarged donor suturing interface	SIE arterial opening with retained femoral-wall patch to common carotid artery, ES; corresponding venous preparation to external jugular vein, EE	2: 1 arterial 1 venous	Preservation of the femoral-wall patch; appropriate recipient arteriotomy; posterior-wall suturing; adaptation of suture spacing to the anastomotic interface	Common endpoints, plus assessment of patch preparation, arteriotomy and posterior-wall technique	Two consecutive complete exercises meeting all common pass criteria before advancement to Stage V
V: Cervical transfer using native SIE vessel ends	Perform anastomosis without the enlarged interface provided by a femoral-wall patch	Native SIE artery to common carotid artery, ES; native SIE vein to external jugular vein, EE	2: 1 arterial 1 venous	Handling of native epigastric vessel ends; precise bites and knot tension; adaptation to donor–recipient size differences; avoidance of luminal narrowing	Common endpoints, plus assessment of native-vessel preparation and anastomotic geometry	Two consecutive complete exercises meeting all common pass criteria constitute completion of the experimental module, followed by supervisor review

## Data Availability

The dataset is available on request from the authors.

## Supplementary Materials

The following supporting information can be downloaded at: https://www.mdpi.com/article/10.3390/medicina62101963/s1, Figure S1: Residual diagnostics for the separate ordinary least-squares regressions of individual anastomosis time on stage number, coded from 1 to 5. Panels A and C show residuals against fitted time for arterial (*n* = 30) and venous (*n* = 26) anastomoses, respectively. Small horizontal offsets separate coincident observations; residual values are unchanged. Panels B and D show the corresponding normal Q–Q plots with reference lines. Regression inference used HC3 heteroscedasticity-consistent standard errors and remained unadjusted for within-animal clustering. These plots describe residual patterns and do not establish independence of observations within animals; Checklist S1: Completed ARRIVE 2.0 reporting checklist.

## Abbreviations

The following abbreviations are used in this manuscript:

SIE	Superficial Inferior Epigastric
EE	End to end
ES	End to side
A	Arterial
V	Venous
