# Supplementary material for: A Stepwise Preclinical Microvascular Training Model Based on the Superficial Inferior Epigastric Vessel Flap in the Rat: A Pilot Study
Source: Medicina (Kaunas). 2026 Oct 11;62(10):1963. doi: 10.3390/medicina62101963 (PMC13707335; doi:10.3390/medicina62101963)
Supplement: Supplementary file 1 [file medicina-62-01963-s001.zip › S1 - Figure S1.pdf]

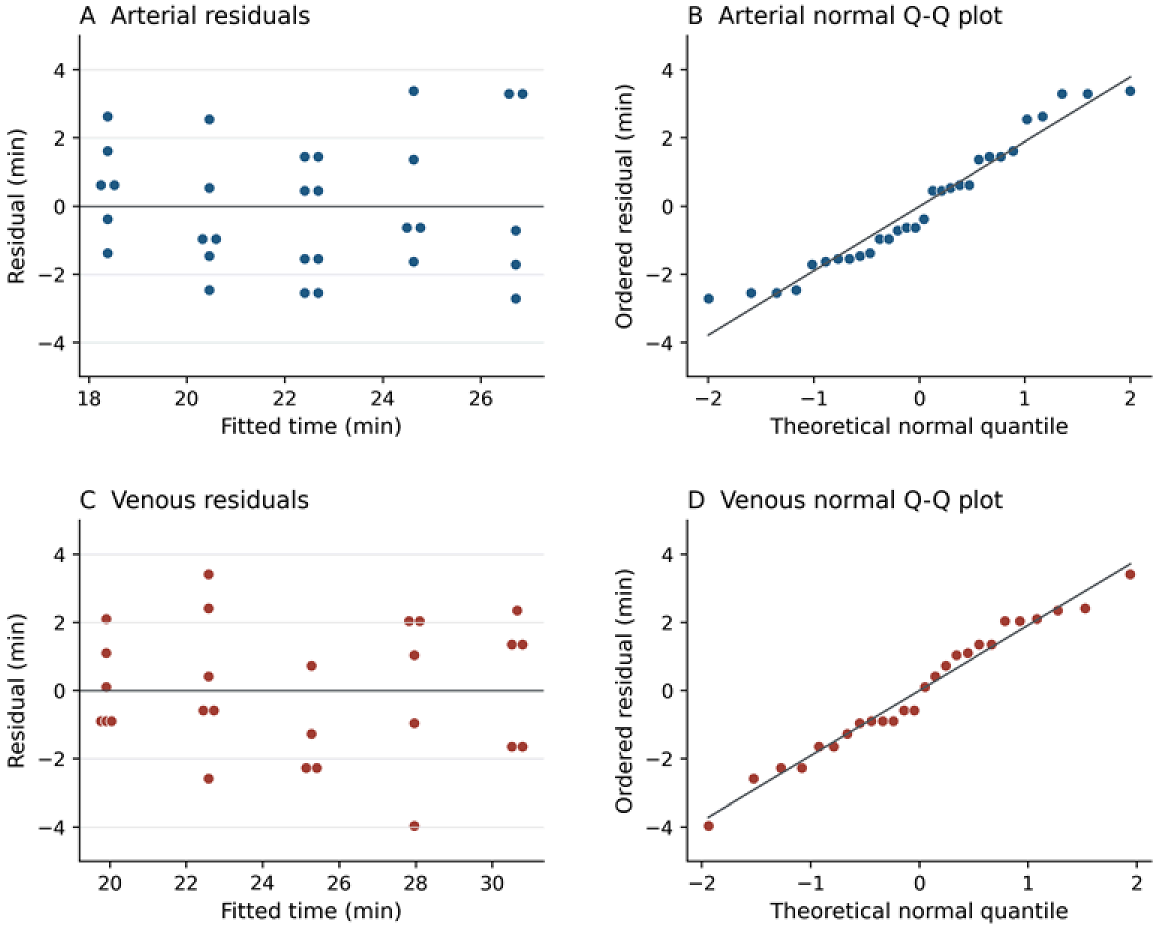

Figure S1. Residual diagnostics for the separate ordinary least-squares regressions of individual anastomosis time on stage number, coded from 1 to 5. Panels A and C show residuals against fitted time for arterial (n = 30) and venous (n = 26) anastomoses, respectively. Small horizontal offsets separate coincident observations; residual values are unchanged. Panels B and D show the corresponding normal Q–Q plots with reference lines. Regression inference used HC3 heteroscedasticity-consistent standard errors and remained unadjusted for within-animal clustering. These plots describe residual patterns and do not establish independence of observations within animals.
